# FIH Regulates Cellular Metabolism through Hydroxylation of the Deubiquitinase OTUB1

**DOI:** 10.1371/journal.pbio.1002347

**Published:** 2016-01-11

**Authors:** Carsten C. Scholz, Javier Rodriguez, Christina Pickel, Stephen Burr, Jacqueline-alba Fabrizio, Karen A. Nolan, Patrick Spielmann, Miguel A. S. Cavadas, Bianca Crifo, Doug N. Halligan, James A. Nathan, Daniel J. Peet, Roland H. Wenger, Alex Von Kriegsheim, Eoin P. Cummins, Cormac T. Taylor

**Affiliations:** 1 Systems Biology Ireland, University College Dublin, Belfield, Dublin, Ireland; 2 School of Medicine and Medical Science, University College Dublin, Belfield, Dublin, Ireland; 3 The Conway Institute, University College Dublin, Belfield, Dublin, Ireland; 4 Institute of Physiology and Zürich Center for Integrative Human Physiology (ZIHP), University of Zürich, Zürich, Switzerland; 5 Cambridge Institute for Medical Research, Department of Medicine, University of Cambridge, Cambridge Biomedical Research Centre, Cambridge, United Kingdom; 6 School of Biological Sciences, University of Adelaide, Adelaide, South Australia, Australia; University of Pennsylvania, UNITED STATES

## Abstract

The asparagine hydroxylase, factor inhibiting HIF (FIH), confers oxygen-dependence upon the hypoxia-inducible factor (HIF), a master regulator of the cellular adaptive response to hypoxia. Studies investigating whether asparagine hydroxylation is a general regulatory oxygen-dependent modification have identified multiple non-HIF targets for FIH. However, the functional consequences of this outside of the HIF pathway remain unclear. Here, we demonstrate that the deubiquitinase ovarian tumor domain containing ubiquitin aldehyde binding protein 1 (OTUB1) is a substrate for hydroxylation by FIH on N22. Mutation of N22 leads to a profound change in the interaction of OTUB1 with proteins important in cellular metabolism. Furthermore, in cultured cells, overexpression of N22A mutant OTUB1 impairs cellular metabolic processes when compared to wild type. Based on these data, we hypothesize that OTUB1 is a target for functional hydroxylation by FIH. Additionally, we propose that our results provide new insight into the regulation of cellular energy metabolism during hypoxic stress and the potential for targeting hydroxylases for therapeutic benefit.

## Abstract 2

The oxygen-dependent asparagine hydroxylase FIH regulates the transcription factor HIF during the cellular response to hypoxia. This study suggests that FIH may also contribute to the hypoxia response by affecting cellular metabolism via altered deubiquitinase targeting.

## Author Summary

Hypoxia is a commonly encountered physiologic and pathophysiologic stress to which mammalian cells have evolved an effective adaptive response. This response is governed by a transcription factor termed the hypoxia-inducible factor (HIF). The mechanisms linking the cellular sensing of oxygen levels to HIF activation have been elucidated and involve oxygen-dependent hydroxylation of HIF on proline and asparagine residues by a family of hydroxylases. A key question that remains unclear is the extent to which oxygen-dependent hydroxylation occurs as a functional post-translational modification outside of the HIF pathway. This is key to developing our understanding of whether hydroxylation is a general regulatory modification or one which has specifically evolved for the regulation of HIF. Here, we demonstrate that the deubiquitinase ovarian tumor domain containing ubiquitin aldehyde binding protein 1 (OTUB1) is a target for functional hydroxylation by the FIH hydroxylase. Hydroxylation of OTUB1 by FIH on asparagine residue N22 results in a restriction in its interactome, leading us to hypothesize a possible role for hydroxylation in substrate targeting. Of interest, interactions of OTUB1 with a number of proteins involved in metabolism are altered upon removal of the hydroxylation site—implicating OTUB1 as a possible link between oxygen sensing and the regulation of metabolism.

## Introduction

Hypoxia is a common feature of the microenvironment in a number of pathophysiologic conditions and represents a significant threat to cellular metabolic homeostasis [1]. Eukaryotic cells have evolved the capacity to rapidly sense changes in intracellular oxygen levels through a family of hydroxylases that confer oxygen-dependence upon the key transcriptional regulator of the adaptive response to hypoxia, termed the hypoxia-inducible factor (HIF) [2,3].

Hydroxylases were first identified as oxygen sensors in the HIF pathway and belong to the Fe(II)- and 2-oxoglutarate-dependent dioxygenase superfamily [4]. These enzymes catalyze the hydroxylation of proteins, in a manner that is dependent on the availability of molecular oxygen (O_2_). Therefore, hydroxylase activity is decreased when O_2_ is low [5]. Four discrete HIF-hydroxylase isoforms have been identified to date, three of which are prolyl-4-hydroxylases (PHD1–3) (Uniprot accession numbers: Q96KS0, Q9GZT9, Q9H6Z9), which regulate the stability of HIFα subunits. In normoxia, the PHDs hydroxylate specific proline residues (P402 and P564 on HIF-1α), which promotes binding of the von Hippel-Lindau (VHL) (Uniprot accession number: P40337) protein followed by formation of an E3 ubiquitin ligase complex, HIFα ubiquitination and subsequent degradation [2]. In parallel with this, a second oxygen-dependent repression of HIF transcriptional activity is regulated by asparaginyl hydroxylation. The asparagine hydroxylase, termed factor inhibiting HIF (FIH) (Uniprot accession number: Q9NWT6), hydroxylates an asparagine residue within HIFα subunits (N803 on HIF-1α), resulting in steric inhibition of its interaction with the transcriptional co-activator p300/CBP, thereby inhibiting HIF-dependent transcription [2]. In hypoxia, when HIFα hydroxylation is reduced, HIFα subunits escape degradation, translocate into the nucleus, bind to the subunit HIF-1β, form a transcriptional complex with p300/CBP and activate gene expression [6].

A number of proteins other than HIF are also sensitive to regulation by hypoxia. However, the mechanism governing their oxygen-sensitivity is less clear [7]. A key question that remains is whether functional hydroxylation is specific for the regulation of HIFα or if other oxygen-sensitive proteins are also regulated by this post-translational modification. While the number of proteins identified as being targets for proline hydroxylation is low [8–10], FIH-dependent asparagine hydroxylation has been demonstrated for a larger group of non-HIF substrates, including ankyrin-repeat domain (ARD)-containing proteins such as tankyrase, notch-1, ASPP2, and IκBα [11–14]. However, the functional consequences of asparagine hydroxylation in general remain less clear [15,16]. Notably, a functional hydroxylation of the ion channel TRPV3 by FIH has recently been reported [17].

FIH homozygous knockout mice demonstrate a metabolic phenotype leading to the proposal that FIH is a key regulator of cellular metabolism [18]. However, a change of HIF activity alone due to FIH knockout could not explain all of the observed metabolic changes [18], indicating HIF-independent mechanisms. Because of this, a key question remaining is whether other FIH target proteins are involved in the observed metabolic phenotype in FIH-deficient mice.

In a previous study, we identified a number of putative hydroxylation substrates in the IL-1β signaling pathway that could account for the inhibition of IL-1β-induced inflammation observed in cells treated with pharmacologic hydroxylase inhibitors [19]. One identified candidate for asparagine hydroxylation was the deubiquitinase (DUB) ovarian tumor domain containing ubiquitin aldehyde binding protein 1 (OTUB1) (Uniprot accession number: Q96FW1) [19]. Interestingly, in a global proteomic analysis, OTUB1 was identified to interact with metabolic regulators [20]. Furthermore, mice deficient in OTUB1 demonstrate a lean body mass phenotype that is reflective of altered metabolic function (http://www.mousephenotype.org/data/genes/MGI:2147616).

In the current study, we provide evidence that N22 of OTUB1 is a bona fide target for enzymatic hydroxylation by FIH as demonstrated by a combination of mass spectrometric techniques and in vitro peptide hydroxylation. Furthermore, site-directed point mutagenesis of N22 enhanced the OTUB1 interactome, particularly with respect to proteins involved in metabolism. Finally, overexpression of mutant OTUB1 resulted in cellular bio-energetic stress (as reflected by enhanced AMP kinase activation) when compared to wild-type (WT) OTUB1, thus indicating a functional role for N22 hydroxylation in terms of regulating cellular metabolism. These data provide a mechanistic link between FIH-dependent hydroxylation of OTUB1 and alterations in cellular metabolism and contribute a further level of understanding to the vital link between cellular oxygen-sensing mechanisms and the control of cellular metabolism.

## Results

### Regulation of Cellular Metabolism by FIH and OTUB1

**Fig 1:**
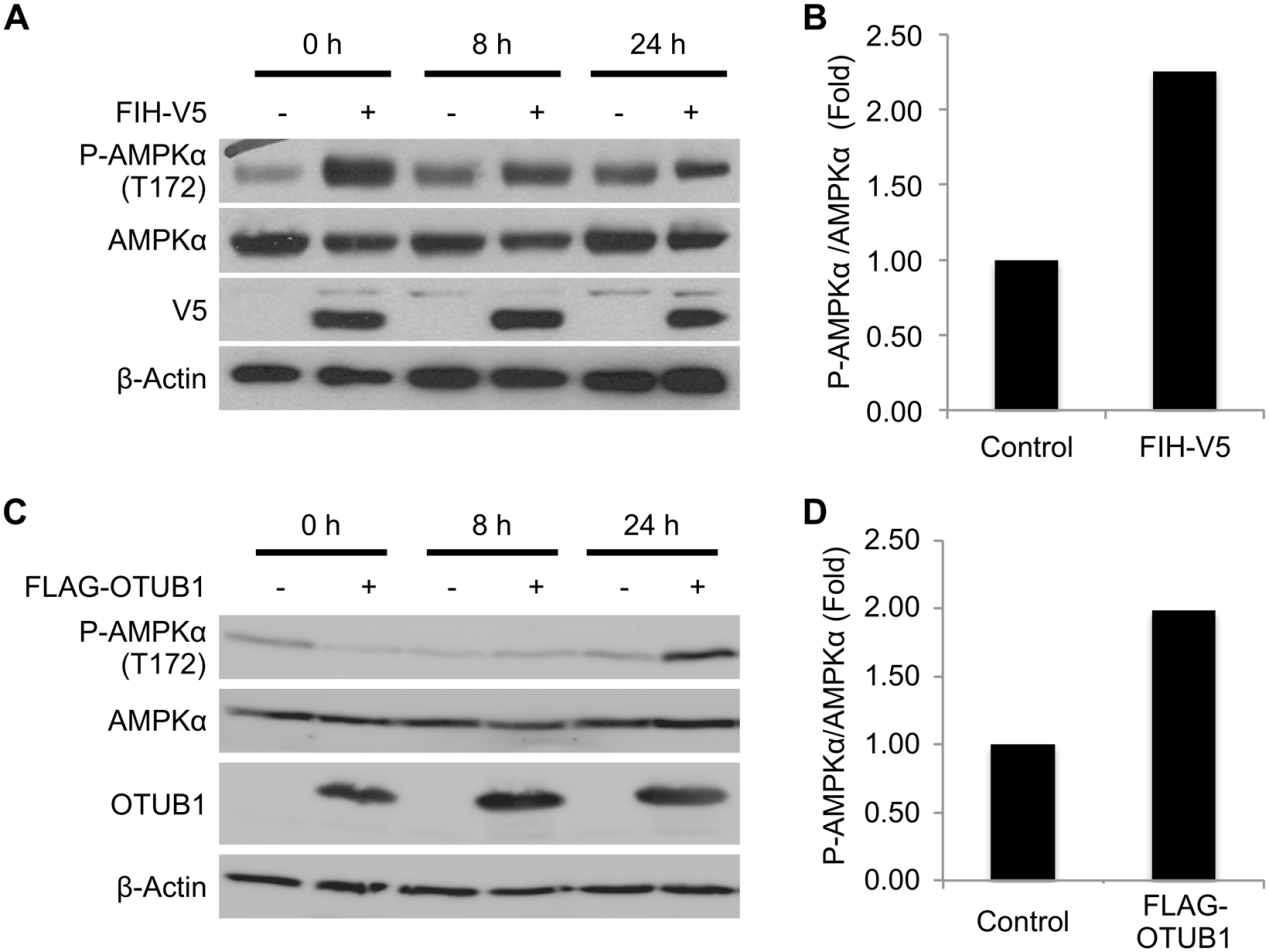
FIH and OTUB1 regulate cellular metabolism. **(A)** Western blot analysis of the impact of FIH overexpression on phosphorylation of AMPKα on T172 in HEK293 cells. Cells were transfected with either an empty vector or FIH-V5 for 24 h prior to media change. Phospho-AMPKα, total AMPKα, β-actin, and FIH-V5 expression were assessed for up to 24 h following the addition of fresh medium by western blot. **(B)** Densitometric analysis of AMPKα phosphorylation at the time point 0 h. **(C)** Analysis of the impact of OTUB1 WT overexpression on phosphorylation of AMPKα (T172) in HEK293 cells. Cells were transfected with either an empty vector or pFLAG-OTUB1 WT for 24 h prior to media change, and phospho-AMPKα, total AMPKα, β-actin, and FLAG-OTUB1 expression were assessed for up to 24 h by western blot. **(D)** Densitometric analysis of AMPKα phosphorylation at the time point 24 h. Data are presented as a representative blot or mean densitometric analysis derived from *n* = 4 independent experiments. The underlying data of panels B and D can be found in S1 Data.

Previous studies have linked homozygous FIH deficiency in mice with a phenotype of disrupted energy metabolism. This is in part reflected by altered phosphorylation of AMPKα, which reflects a change in the cellular AMP:ATP ratio and therefore can be used as a surrogate marker of cellular metabolic stress. The molecular mechanisms underpinning this alteration in AMPKα phosphorylation remain unknown, but appear to be independent of the prototypic FIH substrate HIF [18]. In order to test whether FIH regulates energy metabolism at the cellular level, HEK293 cells were transfected with FIH-V5 for 24 h and cell extracts were generated either prior to the addition of fresh medium or 8–24 h later. Overexpression of FIH led to an increase in phosphorylation of AMPKα compared to cells transfected with an empty vector (Fig 1A and 1B). This effect was most prominent prior to the addition of fresh medium. Overexpression of the catalytically dead mutant FIH H199A did not change AMPKα phosphorylation (S1A and S1B Fig). These data support previous work linking FIH hydroxylase activity to the regulation of metabolic processes in cells.

In a previous global screen, we identified the DUB OTUB1 as a putative new substrate for asparagine hydroxylation by FIH [19]. Furthermore, mice deficient in OTUB1 demonstrate a phenotype that is consistent with altered metabolism (http://www.mousephenotype.org/data/genes/MGI:2147616). Also, OTUB1 was shown to interact with proteins involved in the regulation of cellular metabolism [20]. In support of this, we found that overexpression of OTUB1 in HEK293 cells was associated with an increase in phosphorylation of AMPKα 24 h following the addition of fresh media (Figs 1C, 1D and S2A). This indicates that the cells were experiencing metabolic stress as reflected by an imbalance in the cellular AMP:ATP ratio (and subsequent AMPK activation) when OTUB1 activity is increased. Furthermore, overexpression of FIH together with simultaneous knockdown of OTUB1 prevented the FIH-dependent increase in AMPKα phosphorylation (S1C, S1D and S2B Figs). Overall, these data led us to hypothesize that OTUB1 hydroxylation by FIH may be a link in the association of FIH with an alteration in cellular metabolism.

### OTUB1 Is Hydroxylated on N22 by Endogenous FIH

**Fig 2:**
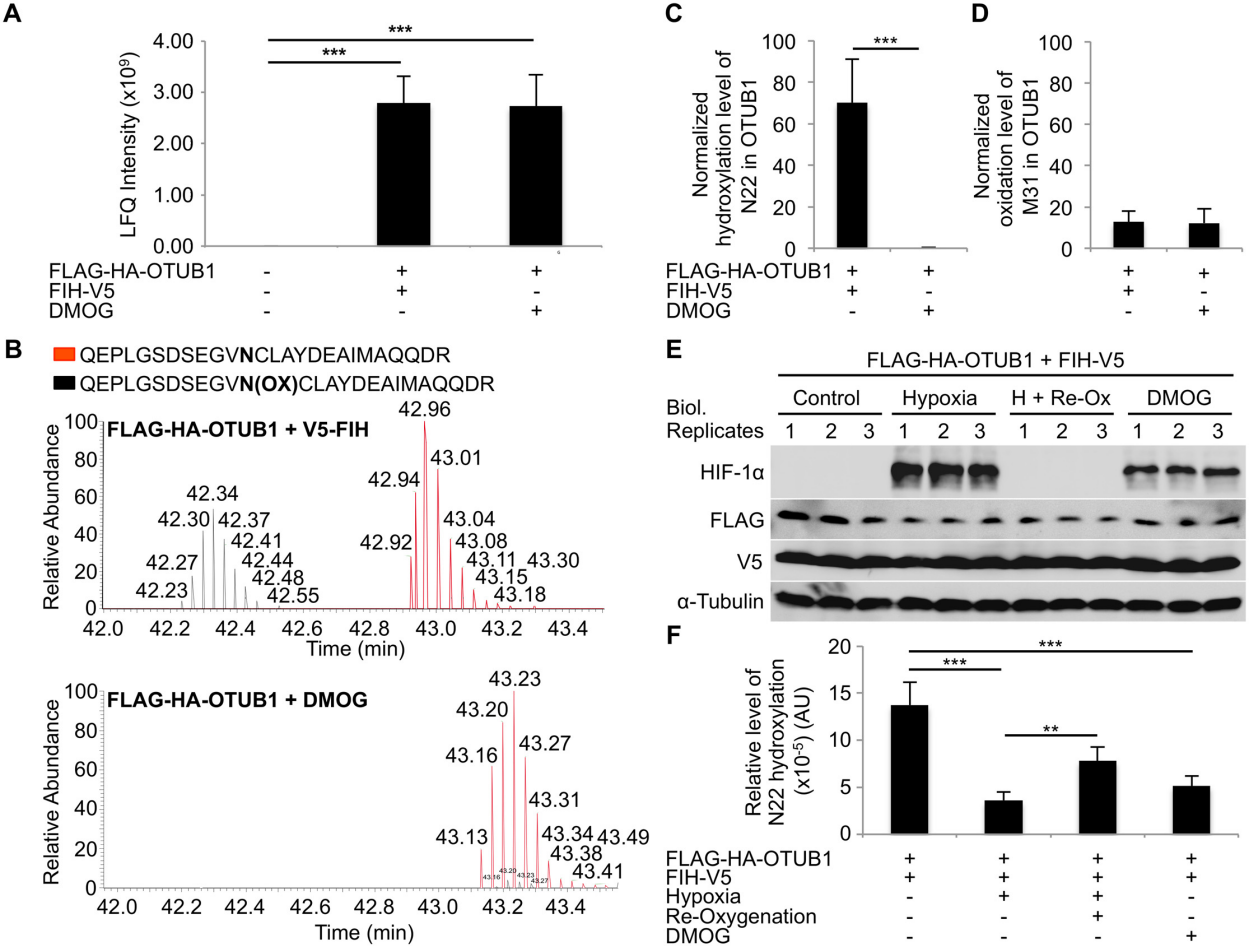
OTUB1 is enzymatically hydroxylated on asparagine 22. HEK293 cells were transfected with either an empty vector (negative control conditions), pFLAG-HA-OTUB1 and pFIH-V5 with 50 μM ascorbate (high asparagine hydroxylation capacity conditions), or pFLAG-HA-OTUB1 and an empty vector with 2 mM DMOG (low hydroxylation capacity conditions). Following FLAG-specific immunoprecipitation samples were analyzed by mass spectrometry. **(A)** Mass spectrometric analysis of the relative amount of immunoprecipitated OTUB1 in each sample. **(B)** Extracted ion chromatogram demonstrating levels of OTUB1 peptides containing hydroxylated and non-hydroxylated asparagine 22 in cells exposed to either high or low hydroxylation capacity conditions. **(C)** Hydroxylation levels of asparagine 22 of OTUB1 in high and low hydroxylation capacity conditions. The detected hydroxylation peptide intensity of each sample was normalized to the amount of non-hydroxylated OTUB1 N22 peptide intensity detected. **(D)** Oxidation levels of methionine 31 in OTUB1 in high and low hydroxylation capacity conditions. The detected oxidation peptide intensity of each sample was normalized to the amount of non-oxidized OTUB1 M31 peptide intensity detected. **(E)** HEK293 cells were transfected with pFLAG-HA-OTUB1 and pFIH-V5 for 24 h prior to an 8 h treatment with 50 μM ascorbate (all samples) in combination with either 1 mM DMOG or incubation in normoxia (control; 21% O_2_) or hypoxia (0.2% O_2_). For instantaneous re-oxygenation, hypoxic media was exchanged with normoxic media and samples were incubated for 1 h in normoxia following the 8 h of hypoxic exposure (H + Re-Ox). Treatment and overexpression efficiency were determined by western blot. **(F)** Hydroxylation levels of OTUB1 N22 of the samples shown in (E) after FLAG-specific immunoprecipitation. The detected hydroxylation peptide intensity of each sample was normalized to the overall amount of total OTUB1 intensity detected. The experiments were performed with three biological replicates and two technical replicates (in the case of mass spectrometric analysis) per sample. Data are represented as mean + standard deviation (SD). ** *p* < 0.01, *** *p* < 0.001 by Student’s *t* test for (A) and (C) and by one-way ANOVA followed by Tukey post test for (F). AU: arbitrary units. The underlying data of panels A, C, D, and F can be found in S1 Data.

In order to determine whether OTUB1 is a bona fide FIH substrate, initially we used mass-spectrometry–based approaches. First, we tested whether OTUB1 is enzymatically hydroxylated (as opposed to this being a spurious chemical oxidation event). To do this, in one set of cells we maximized the asparagine hydroxylation capacity by overexpressing FIH along with OTUB1. A second set of cells also overexpressing OTUB1 (but not FIH) were treated with the pan-hydroxylase inhibitor DMOG to minimize hydroxylation capacity. Successful overexpression of OTUB1 is demonstrated by immunoprecipitation and quantitative mass spectrometric analysis (Fig 2A). We next investigated the hydroxylation status of immunoprecipitated OTUB1. Fig 2B shows an extracted ion chromatogram of an OTUB1 peptide containing the N22 residue. Mass spectrometric analysis revealed that the peptides with the shorter retention time corresponded with the hydroxylated form of this peptide. Decreased retention time has previously been demonstrated to be associated with FIH-dependent hydroxylation of peptides [11]. Tandem mass spectrometry (MS/MS) analysis of immunoprecipitated OTUB1 peptide demonstrated N22 hydroxylation in the sample where FIH was also overexpressed but not in the cells treated with DMOG (Figs 2B, 2C and S3). Importantly, the oxidation/hydroxylation of M31 (reflecting a nearby spurious oxidation event) in the same samples was independent of enzymatic hydroxylase activity (Fig 2D). Methionine residues are highly susceptible to spurious oxidation [21].

In order to investigate if the observed OTUB1 N22 hydroxylation was regulated by physiologically relevant changes in the cellular microenvironment, we incubated HEK293 cells overexpressing both OTUB1 and FIH in 0.2% oxygen for 8 h with and without subsequent re-oxygenation at 21% oxygen for one additional hour. The analysis of the OTUB1 N22 hydroxylation levels by mass spectrometry showed a significant reduction of OTUB1 N22 hydroxylation in hypoxia which was significantly reversed by re-oxygenation (Fig 2E and 2F). DMOG-dependent inhibition of hydroxylases led to a similarly reduced OTUB1 N22 hydroxylation level as hypoxia (Fig 2F). Of note, the DMOG-dependent inhibition of OTUB1 hydroxylation was partly reversed by FIH overexpression when compared to the effect of DMOG without FIH overexpression (Fig 2C and 2F). Nutrient starvation for 8 h with and without re-introduction of nutrients following for one additional hour also down-regulated OTUB1 N22 hydroxylation, although to a lesser degree than hypoxia (S4A Fig). Overall, these data strongly support the contention that N22 of OTUB1 is a bona fide substrate for enzymatic hydroxylation by FIH and that this is regulated by changes in the cellular microenvironment such as hypoxia.

**Fig 3:**
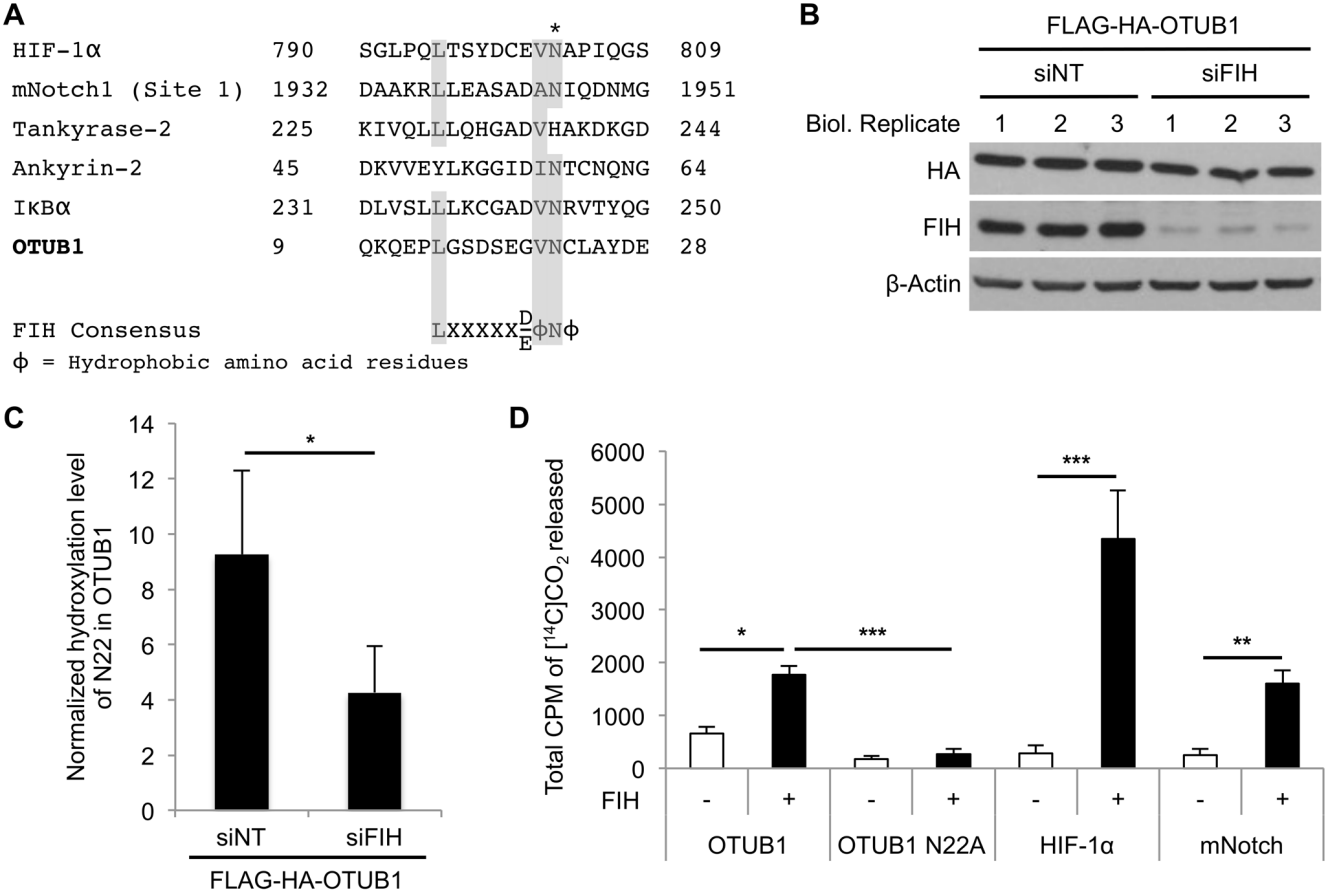
FIH hydroxylates OTUB1 on asparagine 22. **(A)** Sequence alignment of OTUB1 with previously identified FIH target proteins reveals that N22 lies within an FIH consensus motif in OTUB1. Amino acid residues that are part of the FIH consensus sequence in human OTUB1 are highlighted in gray. The FIH-targeted N22 residue of human OTUB1 is highlighted with “*”. **(B)** Western blot analysis demonstrating effective knockdown of FIH in HEK293 cells. **(C)** Hydroxylation levels of OTUB1 N22 of the samples shown in (B) after FLAG-specific immunoprecipitation. The detected hydroxylation peptide intensity of each sample was normalized to the amount of non-hydroxylated OTUB1 N22 peptide intensity detected. **(D)** In vitro CO_2_ capture assay for MBP-hFIH-1-catalyzed hydroxylation-coupled stoichiometric release of ^14^CO_2_ from [1-^14^C]-2-oxoglutarate with synthesized peptides as FIH substrates containing demonstrated hydroxylation sites of HIF-1α, NOTCH and the putative hydroxylation site (N22) of OTUB1. An OTUB1 peptide containing a N22A point mutation was used as control for an N22-specific reaction. The experiment described in (C) was performed with three biological replicates and two technical replicates per sample and data are presented as mean + SD; * *p* < 0.05 by Student’s *t* test. Data presented in (D) as mean + SD of *n* = 3 experiments; * *p* < 0.05, ** *p* < 0.01, *** *p* < 0.001 by one-way ANOVA followed by Tukey post test. The underlying data of panels C and D can be found in S1 Data.

We next investigated whether OTUB1 hydroxylation on N22 is FIH-dependent. Alignment of the amino acid sequence around N22 of OTUB1 with known FIH substrates and a recently published consensus sequence for FIH target proteins revealed that N22 of OTUB1 lies within a motif highly similar to the consensus sequence (Fig 3A) [22]. Protein sequence alignments indicated that the OTUB1 consensus sequence is evolutionary conserved within mammals (S4B Fig). We next overexpressed FLAG-HA-OTUB1 in HEK293 cells and either treated these cells with non-targeting siRNA (siNT) or siRNA targeting endogenous FIH (siFIH) (Figs 3B and S2C). OTUB1 was immunoprecipitated and the hydroxylation status of N22 was analyzed by quantitative mass spectrometry. The hydroxylation of N22 was decreased in cells treated with FIH siRNA supporting the concept that N22 of OTUB1 is a target for endogenous enzymatic FIH-dependent hydroxylation (Fig 3C). Furthermore, to demonstrate that N22 is directly hydroxylated by FIH, FIH activity was measured in an in vitro CO_2_ capture assay using purified FIH and either wild-type OTUB1 peptides containing N22 or OTUB1 peptides in which N22 was replaced by an alanine residue (N22A). Using this assay, which measures the turnover of 2-oxoglutarate into succinate and CO_2_ by FIH, we found a significant increase in FIH activity in the presence of wild-type but not mutant OTUB1 peptide (Fig 3D). Taken together, these data demonstrate that N22 of OTUB1 is a bona fide substrate for enzymatic hydroxylation by FIH.

### Mutation of the OTUB1 Hydroxylation Site Regulates its Interactome

**Fig 4:**
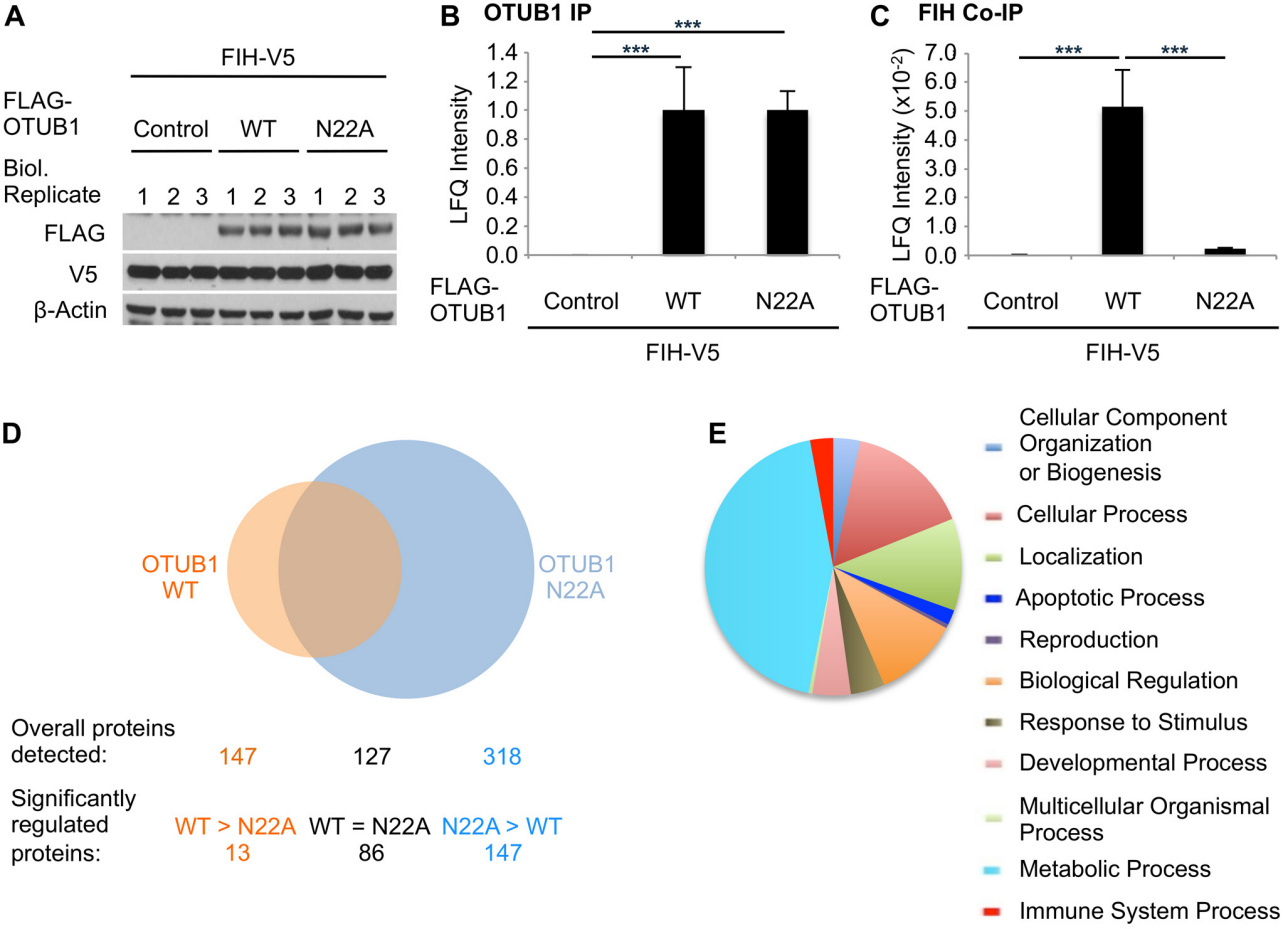
Point mutation of asparagine 22 to alanine regulates the OTUB1 interactome. HEK293 cells were transfected in biological triplicates with pFIH-V5 and empty vector (control), pFLAG-OTUB1 WT, or pFLAG-OTUB1 N22A for 24 h. Following FLAG immunoprecipitation, the precipitates were analyzed for OTUB1-associated proteins by mass spectrometry. **(A)** Western blot analysis of overexpression levels of OTUB1 WT and N22A. **(B)** Relative amount of immunoprecipitated OTUB1 in each sample. **(C)** Relative amount of FIH co-precipitated with OTUB1 in each sample (normalized to precipitated OTUB1 levels). **(D)** Number of interacting proteins enriched in FLAG-OTUB1 WT or N22A over control. “Significantly regulated proteins” indicates the number of proteins being significantly different between FLAG-OTUB1 WT and N22A as indicated. Peptide sequences that were assigned to several proteins were counted as a single interaction. **(E)** Gene Ontology analysis for biological processes of proteins significantly enriched and increased by at least 2-fold in FLAG-OTUB1 N22A over FLAG-OTUB1 WT. The experiment was performed with three biological replicates and two technical replicates per sample. (B), (C) Data presented as mean + SD. *** *p* < 0.001 by Student’s *t* test. (D), (E) The analyses were performed using Panther database (www.pantherdb.org).

We next investigated possible functional consequences of OTUB1 hydroxylation on N22. To do this, we used the N22A mutant in order to prevent FIH-dependent hydroxylation at this site. HEK293 cells were co-transfected with either FLAG-OTUB1 WT or FLAG-OTUB1 N22A, along with FIH (in order to maximize hydroxylation capacity; Fig 4A). Following immunoprecipitation of OTUB1 (demonstrated in Fig 4B) we identified the OTUB1 WT and the OTUB1 N22A interactomes by mass spectrometry. Initially, we confirmed the interaction of both OTUB1 WT and OTUB1 N22A with six previously described OTUB1 interacting proteins (S5 Fig) [20,23–27]. We next used the interaction of FIH with OTUB1 as additional positive control [19] and confirmed this interaction in the case of OTUB1 WT in the interactome experiment and also subsequently by western blot analysis (Figs 4C and S5G). Interestingly, this interaction was greatly reduced with OTUB1 N22A, indicating a key role for this residue in the interaction between FIH and OTUB1 (Figs 4C and S5G).

Qualitative analysis revealed that 147 proteins were associated with OTUB1 WT, while 318 proteins were associated with OTUB1 N22A (Fig 4D). Of the OTUB1 interacting proteins, 127 were associated with both OTUB1 WT and OTUB1 N22A, indicating that the core interactome is not affected by mutation of N22. However, when we compared the OTUB1 WT and the OTUB1 N22A interactomes, we found that while just 13 proteins were enriched in their association with OTUB1 WT over OTUB1 N22A, 147 proteins were enriched in their association with OTUB1 N22A over OTUB1 WT (Fig 4D). There were 86 proteins that had equivalent levels of interaction with both OTUB1 WT and N22A. This indicates that loss of hydroxylation on N22 leads to more than a doubling of the number of proteins in the OTUB1 interactome through the recruitment of new binding partners. Of note, the N22A point mutation of OTUB1 did not change its deubiquitinase activity, which indicates that this mutation does not significantly alter protein structure (as catalytic activity is retained) (S6 Fig). Based on these data, we hypothesize that hydroxylation of N22 on OTUB1 profoundly alters its interaction with other proteins and is therefore likely of functional consequence.

Ontological analysis of the proteins differentially associated with OTUB1 WT and OTUB1 N22A using the Panther database (www.pantherdb.org) revealed proteins associated with multiple biological processes (S7A and S7B Fig). However, metabolism-associated proteins were most highly represented, which is in agreement with previously published data for wild-type OTUB1 by Sowa et al., demonstrating that OTUB1 interacts with metabolic regulators [20]. Furthermore, OTUB1 N22A had increased numbers of metabolism-associated proteins when compared to OTUB1 WT (Fig 4E and S1 Table) indicating that loss of N22 hydroxylation may impact upon interaction between OTUB1 and multiple proteins important in the regulation of metabolism. In summary, we demonstrate that N22A mutation of OTUB1 profoundly alters its physical interactome. Of note, proteins associated with metabolic processes are heavily represented in this cohort.

### OTUB1 Wild Type and OTUB1 N22A Differentially Regulate Phosphorylation of AMPKα during Nutrient Starvation

**Fig 5:**
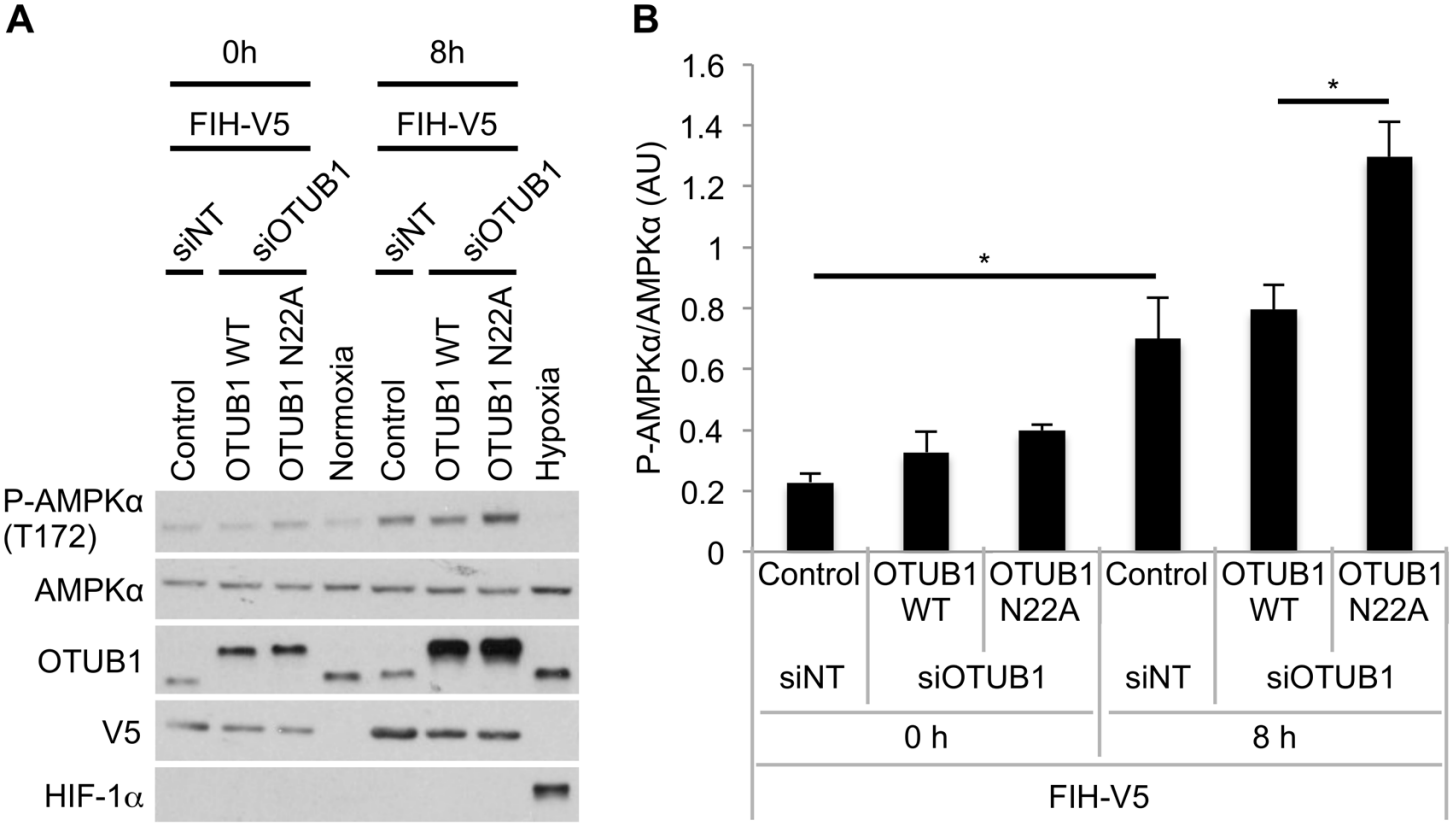
Mutation of the OTUB1 N22 hydroxylation site impacts on AMPKα phosphorylation in response to glucose, pyruvate, and glutamine starvation. **(A)** HEK293 cells were transfected with either siNT or siOTUB1 for 24 h prior to transfection with pFIH-V5 and either empty vector control, pFLAG-OTUB1 WT or pFLAG-OTUB1 N22A for additional 24 h. Normal culture media was changed to media without glucose, pyruvate and glutamine and the levels of phosphorylation of AMPKα were analyzed at the indicated time points. For the exposure to hypoxia, cells were incubated in 1% O_2_ (normoxia: 21% O_2_) for the indicated time point. **(B)** Densitometric analysis of P-AMPKα levels at 0 h and 8 h. Data was normalized to total AMPKα. Data is presented as mean + standard error of the mean (SEM) of *n* = 3 independent experiments. * *p* < 0.05 by one-way ANOVA followed by Tukey post test. The underlying data of panel B can be found in S1 Data.

We next investigated the impact of N22A mutation of OTUB1 on FIH-dependent regulation of cellular metabolism under conditions of energy starvation in cultured cells. Simultaneous glucose, glutamine, and pyruvate deprivation caused an increase in the phosphorylation of AMPKα likely as a result of ATP depletion (Fig 5A and 5B). Cells overexpressing both wild type OTUB1 and FIH (to maximize OTUB1 hydroxylation) showed similar levels of AMPKα activity as control cells, however, cells overexpressing both FIH and N22A mutated OTUB1 (to minimize OTUB1 hydroxylation) demonstrated robustly increased phosphorylation of AMPKα. These data are consistent with our hypothesis that FIH-dependent N22 hydroxylation of OTUB1 contributes to the regulation of cellular metabolism by FIH.

### OTUB1 N22 Hydroxylation Does Not Affect its Protein Stability

**Fig 6:**
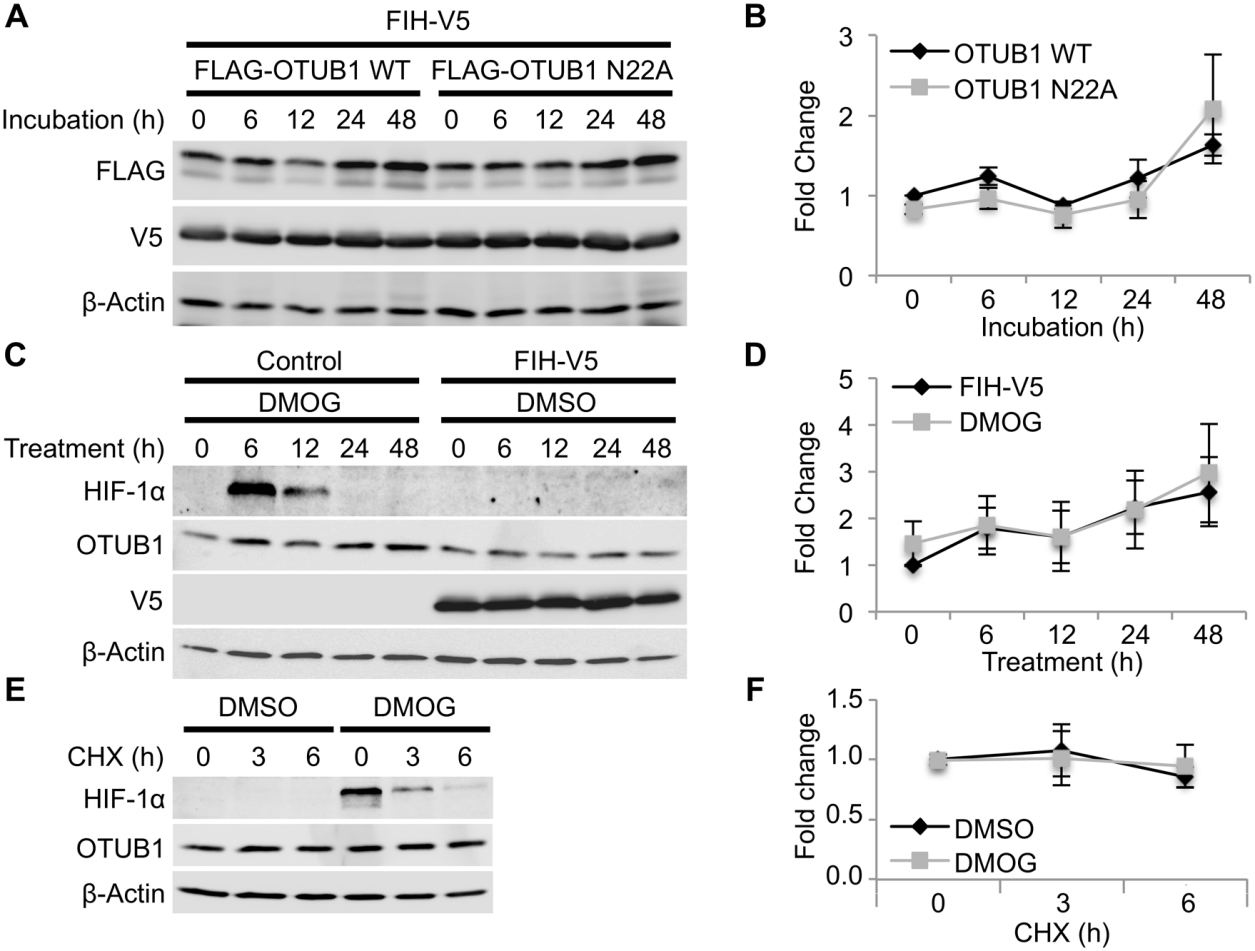
OTUB1 N22 hydroxylation does not impact on OTUB1 protein level and half-life. **(A)** HEK293 cells stably expressing shOTUB1 (targeting the 3′UTR to diminish endogenous OTUB1 protein) and FLAG-OTUB1 WT or N22A were transiently transfected with FIH-V5 for 24 h followed by western blot analysis of OTUB1 WT and N22A protein levels at the indicated time points. **(B)** Densitometric analysis of OTUB1 WT and N22A protein levels. OTUB1 protein levels were normalized to β-actin loading control. **(C)** HEK293 wild-type cells were transiently transfect with either control plasmid or pFIH-V5 (to maximize OTUB1 hydroxylation) for 24 h and subsequently treated with either 1 mM DMOG (to prevent OTUB1 hydroxylation) or DMSO for the indicated time points. Endogenous OTUB1 protein levels were analyzed by western blot. **(D)** Densitometric analysis of endogenous OTUB1 protein levels. OTUB1 protein levels were normalized to β-actin loading control. **(E)** HEK293 wild-type cells were pre-treated with DMSO or 1 mM DMOG (to inhibit OTUB1 hydroxylation) for 16 h prior to the treatment with 50–100 μg/ml cycloheximide (CHX) for the indicated time points. Endogenous OTUB1 protein levels were analyzed by western blot. **(F)** Densitometric analysis of endogenous OTUB1 protein levels. OTUB1 protein levels were normalized to β-actin loading control. Data are presented as representative blot or as mean ± SEM of *n* = 3 independent experiments. The underlying data of panels B, D, and F can be found in S1 Data.

We next investigated the impact of the hydroxylation of N22 on the OTUB1 protein. We considered a potential change of OTUB1 protein levels and its half-life due to the hydroxylation of N22 similar to the described regulation of the HIF-1α protein by prolyl hydroxylation. We therefore established HEK293 cells stably overexpressing FLAG-OTUB1 WT or N22A, which, at the same time, also carried a stably integrated shRNA targeting the 3′UTR of OTUB1 to diminish endogenous OTUB1 protein levels (S8 Fig). We transiently transfected these cells with FIH-V5 to maximize FLAG-OTUB1 WT hydroxylation and analyzed OTUB1 WT and N22A protein levels for up to 48 h by western blot. No significant change between the protein levels of OTUB1 WT and OTUB1 N22A was observed (Fig 6A and 6B). We next investigated if endogenous OTUB1 protein levels change in response to an alteration of N22 hydroxylation levels. We transiently transfected HEK293 cells with either empty vector (control) or FIH-V5 and treated the control cells with DMOG and the FIH overexpressing cells with DMSO. This experimental set up was similar to the experiment performed in Fig 2A–2D, which lead to maximally hydroxylated N22 of OTUB1 in the FIH overexpressing sample and to diminished hydroxylation of N22 in the DMOG-treated sample. We then analyzed endogenous OTUB1 protein levels in a time course for up to 48 h by western blot. No difference was observed between maximally hydroxylated OTUB1 and minimally hydroxylated OTUB1 protein levels (Fig 6C and 6D). In order to investigate the half-life of OTUB1 depending on its hydroxylation status, HEK293 cells were incubated with DMOG for 16 h prior to the treatment with cycloheximide (CHX) to inhibit protein synthesis. Within a time frame of 6 h CHX treatment, in which HIF-1α protein levels significantly decreased, no change in OTUB1 protein levels were observed (Fig 6E and 6F). Overall, these data demonstrate that the hydroxylation of OTUB1 at N22 does not impact on OTUB1 protein stability.

## Discussion

Hypoxia is a common feature of a number of diseases in which metabolism is significantly altered, including chronic inflammation and cancer. The mechanisms by which hypoxia-dependent alterations in metabolism occur in such disease states have important implications for disease development and potential targets for future therapeutic intervention. In this study, we provide new insight into the regulation of metabolism by hypoxia, which is mediated through the DUB OTUB1.

The identification of HIF as a ubiquitous master regulator of the cellular adaptive response to hypoxia and its oxygen-dependent regulation by 2-oxoglutarate-dependent hydroxylases were key discoveries in our developing understanding of the oxygen-sensing mechanisms which operate in eukaryotic cells [21,28–33]. Because several pathways apart from HIF also demonstrate sensitivity to hypoxia, it was initially anticipated that post-translational hydroxylation would be a common modification resulting in the conferral of oxygen sensitivity on multiple targets. However, while it appears that hydroxylation is indeed a common protein modification, understanding the functional role of this outside of the HIF pathway has remained elusive.

Functional proline-hydroxylation of non-HIF proteins by the HIF prolyl hydroxylases has been proposed for a limited number of proteins, including FOXO3a, CyclinD1, ATF-4, and IKKβ [8–10,34,35]. However, asparagine hydroxylation by FIH appears to be a more commonly observed modification and has been clearly demonstrated for multiple non-HIF proteins, including several ARD-containing proteins such as tankyrase, notch-1, and IκBα [11–13]. However, the functional impact of this on cellular signaling pathways (if any) remains unclear. Therefore, the identification of new functional hydroxylation events is of key importance in developing our understanding of this oxygen-sensitive, post-translational modification.

It has recently been demonstrated that mice that are homozygously deficient in FIH demonstrate a metabolic phenotype characterized by (for example) reduced body weight, elevated metabolic rate, and hyperventilation [18]. In these studies, the cellular bioenergetic status was assessed by measurement of the activation of AMPK, a key gauge of cellular metabolic stress which becomes activated when ATP is depleted and the AMP:ATP ratio increases. Because these mice do not display a phenotype consistent with activated HIF, it appears that the mechanisms underpinning the metabolic phenotype are at least in part independent of the HIF pathway and depend upon other FIH-dependent pathways [18]. In this study, we identified OTUB1 to be a new FIH substrate that may be important in the regulation of cell metabolism (as also reflected by altered AMPK activation) and, as such, may provide mechanistic insight into the metabolic phenotype observed in the FIH knockout mouse. Of note, wild-type OTUB1 has been reported to interact with metabolic regulators [20]. Furthermore, while homozygous deletion of OTUB1 results in early lethality, mice heterozygously deficient in OTUB1 were reported to display a metabolic phenotype characterized by decreased lean body mass (http://www.mousephenotype.org/data/genes/MGI:2147616) [36–40]. Taken together, these data suggest the possibility that OTUB1 hydroxylation may at least in part provide a molecular explanation for some aspects of the observed phenotype in FIH-deficient mice.

Previous work has demonstrated a profoundly anti-inflammatory effect of pan-hydroxylase inhibitors (which have inhibitory activity against both PHDs and FIH) in multiple models of intestinal inflammation [41]; however, the full mechanism underpinning this remains unclear. Altered metabolism has recently been demonstrated to be a key regulator of inflammation [42]. Therefore, a possible contributory mechanism for the anti-inflammatory activity of hydroxylase inhibitors is through altered FIH-dependent hydroxylation of OTUB1, leading to differential metabolism at inflamed sites.

**Fig 7:**
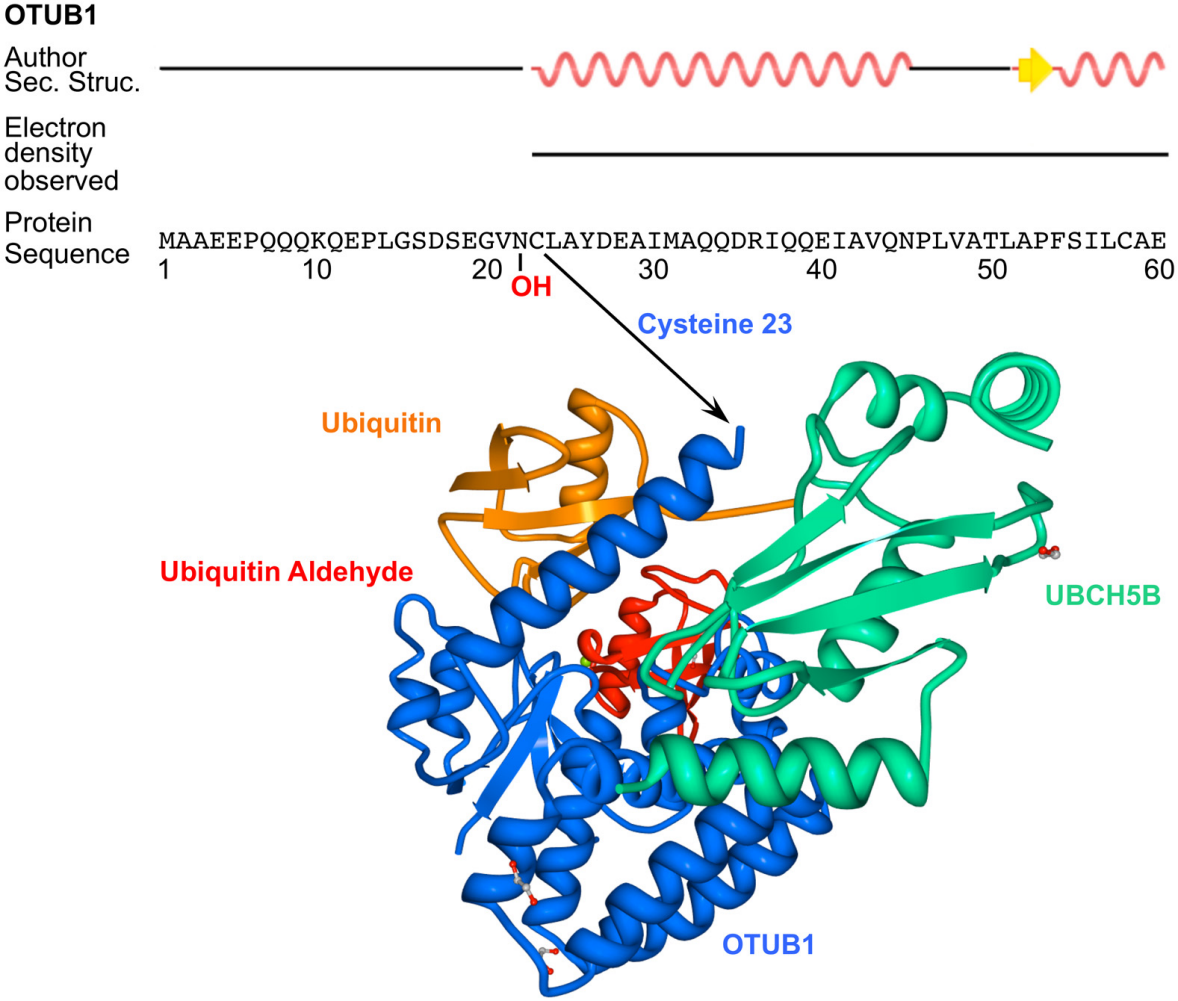
Crystal structure of OTUB1 in complex with UBCH5B, ubiquitin and ubiquitin aldehyde. Indication of the position of the FIH-targeted asparagine 22 of OTUB1 in its secondary, tertiary, and quarternary structure. The image depicts the secondary structure annotation and the crystal structure of a human/*Caenorhabditis elegans* OTUB1 hybrid (containing the N-terminal 45 amino acids of human OTUB1 and the OTU domain of *C*. *elegans* OTUB1) in complex with human UBCH5B (containing a C85S mutation), human ubiquitin and human ubiquitin aldehyde. Structural two-dimensional images and data of the three-dimensional structures were downloaded from RCSB PDB (http://www.rcsb.org/) (PDB ID: 4LDT) and the three-dimensional image was created using Protein Workshop [48,49]. The original data was published by Wiener et al., 2013 [26]. Electron density was not observed for amino acids 1–22 of the N-terminus because these residues were disordered. Author Sec. Struc. = Secondary structure annotation approved by depositor of structure.

In our study, we found that N22, the site of OTUB1 hydroxylation by FIH, is located in a region of the protein that may be key to determining its activity. OTUB1 is unusual in that it hydrolyzes specifically K48 ubiquitin bonds but also inhibits the formation of K63 and K48 ubiquitin chains via a non-canonical, non-catalytic function through inhibition of E2 ubiquitin ligase activity [43–45]. In the OTUB1 apoenzyme, the residues N-terminal to the OTU catalytic domain are disordered (approximately amino acids 1 to 45), whereas upon binding of both distal ubiquitin and an E2 ubiquitin-conjugating enzyme, for example UBCH5B, the folding of a significant portion of the tail becomes stabilized as a structured alpha helix (amino acids 23 to 44) (Fig 7) [26,27,43]. N22 is located at the junction of the α-helix and the remaining unstructured region (amino acids 1 to 22). This is similar to the C-terminal transactivation domain (CAD) of HIF-1α, which is disordered when it is unbound but forms three distinct α-helices upon binding to CBP/p300, of which one helix includes N803, the HIF-1α asparagine residue targeted for hydroxylation by FIH [46,47]. In an attempt to investigate the implications of the hydroxylation of OTUB1 at this key hinge region, we found no impact of OTUB1 hydroxylation on OTUB1 protein levels or half-life (Fig 6). Also, OTUB1 enzymatic activity was unaffected by the N22A point mutation (S6 Fig). Therefore, a direct regulation of the OTUB1 interactome by the N22 hydroxylation resulting in differential substrate targeting seems likely. Consistent with this concept, protein:protein interactions have previously been demonstrated to be directly modified by asparagine hydroxylation in the HIF pathway. FIH-dependent hydroxylation of N803 disrupts HIF-1α interaction with the transcriptional co-activators p300/CBP regulating HIF-1α-dependent trans-activation of gene expression. Ongoing studies are investigating whether hydroxylation of wild-type OTUB1 at N22 impacts on its (non-)canonical activity.

In summary, in this study we provide evidence that OTUB1 is a target for functional hydroxylation by FIH. We propose that this modification may have important implications for the regulation of cellular metabolism by changing OTUB1 substrate targeting under conditions of hypoxia, such as those that occur during ischemia, chronic inflammation, and tumor growth.

## Materials and Methods

### Cell Culture and Transient Transfection

Human embryonic kidney cells (HEK293) were cultivated under standard conditions and used for all experiments presented. Standard cell culture media was DMEM media containing 4.5 g/l glucose, sodium pyruvate and L-glutamine. As media for nutrient starvation experiments DMEM media without glucose, sodium pyruvate or L-glutamine was used. For the transient transfection of both siRNAs and plasmids Lipofectamine 2000 reagent (Invitrogen) was used according to the manufacturer’s description.

### Plasmids, siRNAs and Peptides

The plasmid encoding FIH-V5 was a kind gift of Dr. Eric Metzen (University of Duisburg-Essen, Essen, Germany), whereas the wild-type FLAG-OTUB1 coding plasmid was generously provided by Dr. Mu-Shui Dai (Oregon Health and Science University, Portland, Oregon, United States) [25]. The FLAG-HA-OTUB1 plasmid was a gift from Dr. Wade Harper (Harvard Medical School, Boston, Massachusetts, US) (Addgene plasmid # 22551) [20]. The plasmid encoding FIH H199A has previously been described [32]. Nontargeting siRNA (siNT) was purchased from Dharmacon (GE Healthcare) (ON-TARGETplus SMARTpool). The siRNA targeting FIH (siFIH) was produced by Eurofins Genomics according to a previously reported sequence [11] (sequence F1). siRNA targeting the 3′UTR of OTUB1 was produced by Eurofins Genomics according to a previously reported sequence [25] (siRNA-4).

### Generation of OTUB1 Mutant by Site-Directed Mutagenesis

The FLAG-OTUB1 N22A mutant was generated with the Quikchange II XL Site-Directed Mutagenesis kit (Agilent technologies) according to the manufacturer’s description, using the plasmid encoding FLAG-OTUB1 wild-type as template. Successful mutation was confirmed by sequencing of the targeted site in the obtained plasmid.

### Western Blot Analysis

Protein concentrations of cell lysates for western blot analysis were determined using the Bio-Rad DC protein assay. Equal amounts of protein were separated by SDS PAGE, transferred to nitrocellulose membranes and detected using anti-OTUB1 antibody (Cell Signaling), anti-β-actin antibody (Sigma), anti-AMPKα antibody (Cell Signaling), anti-phospho-AMPKα (Thr172) antibody (Cell Signaling), anti-FIH antibody (Abcam), anti-α-tubulin antibody (Santa Cruz), anti-FLAG antibody (Sigma), anti-V5 antibody (Invitrogen), or anti-HA antibody (Roche).

### In Vitro Hydroxylation Assay

Peptide hydroxylation was assayed by the hydroxylation-coupled decarboxylation of [1-^14^C]-2-oxoglutarate by human FIH (hFIH) as described previously [50]. Each 40 μl reaction contained 3.5 μM MBP-hFIH, 625 μM peptide substrate, 300 μM FeSO_4_, 40 μM 2-oxo[1-^14^C]glutarate (40,000 dpm), 4 mM ascorbate, 500 μM dithiothreitol, 0.4 mg bovine serum albumin, and 50 mM Tris-HCl (pH 7.0), and was incubated at 37°C for 60 min. Filters were dried, UltimaGold XR scintillatant added, and counted on a MicroBeta 2450 (Perkin Elmer).

### Immunoprecipitation and Mass Spectrometric Analysis

Immunoprecipitation was carried out as previously described [19]. Briefly, cell lysates were incubated with the antibody-coupled beads anti-FLAG M2 affinity gel (Sigma) at 4°C for 1 h. Subsequently, the agarose beads were washed twice with lysis buffer (1% Triton X-100, 20 mM Tris-HCl (pH 7.5), 150 mM NaCl, 1 mM MgCl_2_) and twice with washing buffer (20 mM Tris-HCl (pH 7.5), 150 mM NaCl, 1 mM MgCl_2_). This was followed by sample preparation for mass spectrometric analysis as previously described [51]. The samples were analyzed by a Q-Exactive mass spectrometer (Thermo Scientific) and searched with MaxQuant. MS/MS spectra were searched against the human UniProt database (www.uniprot.org). Variable modifications included (MYWNDEPK) hydroxylation/oxidation. For a more detailed description, see Scholz et al. 2013 [19]. The mass spectrometry proteomics data of the OTUB1 hydroxylation experiments have been deposited to the ProteomeXchange Consortium [52] via the PRIDE partner repository with the dataset identifier PXD002103.

### Functional Analysis of OTUB1 Wild-Type and N22A Mutant Interactome Datasets

The datasets of OTUB1 WT and OTUB1 N22A mutant co-precipitated proteins obtained by mass spectrometric analysis were first filtered for significant enrichment of proteins over control. Proteins were only considered as part of the interactome when the average of the six datasets obtained (three biological and two technical replicates) was at least 2-fold different to the negative control and, additionally, when this difference was statistically significant (Student’s *t* test was applied). In addition, the obtained lists were analyzed for differences between the wild-type and mutant interactome. Proteins were only considered to be changed between these two groups when the difference was at least 2-fold and when this difference was statistically significant. These analyses were carried out with Excel (Microsoft). The PANTHER database (www.pantherdb.org) was used for functional annotation of the obtained lists of proteins and to cluster the proteins according to the assigned gene ontology terms [53,54]. The protein interactions from this publication have been submitted to the IMEx (http://www.imexconsortium.org) consortium through IntAct [55] and assigned the identifier IM-23897. For some identified peptides in this experiment, it was not possible to assign the sequence to one specific protein. These results will not be shown in the IntAct database but are available in S2 Data.

### FLAG-OTUB1 Deubiquitinase Assay

1.5 x 10^8^ HEK293 cells stably expressing empty vector (control), FLAG-OTUB1 WT or N22A were harvested by trypsinisation, washed 1x in cold PBS and lysed in 1 ml 1x TBS plus 1% NP40, 5 mM MgCl_2_, 1 mM PMSF and 1x Roche cOmplete EDTA-Free Protease Inhibitor Cocktail per sample on ice for 30 min. Lysates were clarified by centrifugation at 14,000 rpm at 4°C for 10 min and pre-cleared by incubating with 12.5 μl Sepharose 6 fast flow resin and 12.5 μl Protein G Dynabeads (prepared according to manufacturer’s instructions) for 30 min at 4°C with rotation. Immunoprecipitation was performed on pre-cleared lysates by adding 25 μl Protein G Dynabeads and 10 μl ANTI-FLAG M2 antibody per sample and incubating for 2.5 h at 4°C with rotation. Beads were then washed 3x in cold TBS + 0.2% NP40 with a final wash in cold TBS before resuspending in 60 μl TBS. Immunoprecipitated FLAG-OTUB1 WT and N22A (bound to Protein G Dynabeads, equivalent to approximately 3 x 10^7^ cells) was incubated with 600 nM K48-tetraubiquitin (K48-Ub_4_) at 37°C. Samples were harvested at 0, 30, and 60 min. Empty vector IP beads were used as a negative control and 26S proteasomes as a positive control for DUB activity. Samples were mixed with SDS loading buffer, heated at 90°C for 5 min and separated on 4%–12% bis-tris gels. Proteins were transferred to PVDF membranes and blocked in PBS/0.2% Tween-20 plus 3% BSA for 1 h at room temperature. Proteins were detected with HRP-conjugated P4D1 anti-ubiquitin antibody and. ANTI-FLAG M2 antibody.

### Generation of Plasmids Encoding GST-OTUB1 WT or N22A for Bacterial Expression

OTUB1 WT and OTUB1 N22A were amplified by polymerase-based chain reaction (PCR) using pFLAG-OTUB1 WT or N22A as template. The sequences were cloned into the entry vector pENTR4 (Invitrogen) using the restriction enzymes NcoI (Thermo Scientific) and XhoI (Thermo Scientific). Subsequently, pDEST15-OTUB1 WT and N22A (vector carrying the GST-tag) were generated using the gateway system (Invitrogen) with the LR clonase II (Invitrogen) according to the manufacturer’s description.

### Bacterial Expression and Purification of GST-OTUB1 WT and N22A

*Escherichia coli* BL21-AI (Invitrogen) were transformed with pDEST15-OTUB1 WT or N22A, respectively, and protein expression was induced by adding 0.2% L-Arabinose for 3h at 37°C. Bacteria were lysed using a Cell Disruptor (TS Series Bench Top, Constant Systems Ltd.) at 35 kpsi in two cycles. Lysates were cleared by ultracentrifugation at 162,000 xg and 4°C for 1 h (Sorvall WX100 Ultracentrifuge) and subsequently affinity purified with Glutathione Sepharose Fast Flow Columns (GSTrap FF, GE Healthcare) in the duo flow system (Bio-Rad). Successful protein expression and purification was verified by Coomassie staining and western blot against OTUB1.

### GST-OTUB1 Dialysis

Purified GST-tagged OTUB1 WT and N22A were dialysed in 20 mM Tris, 150 mM NaCl, 1 mM DTT to remove GSH. Samples were loaded into SnakeSkin dialysis tubing, sealed and incubated in dialysis buffer at 4°C overnight with gentle stirring. Samples were transferred to fresh buffer for a further 1 h at 4°C. Final protein concentration was assayed using a Nanodrop 2000 spectrophotometer and checked by running equal concentrations of GST-OTUB1 WT and N22A on a 10% SDS PAGE gel and staining with Coomassie brilliant blue.

### GST-OTUB1 Deubiquitinase Assays

Purified GST-OTUB1 WT and N22A were incubated with 600 nM K48-Ub_4_ at 37°C. Samples were harvested at 0, 30, and 60 min. K48-Ub_4_ alone was used as a negative control. Samples were mixed with SDS loading buffer, heated at 90°C for 5 min and run on 4%–12% bis-tris gels. Proteins were transferred to PVDF membranes and blocked in PBS/0.2% Tween-20 plus 3% BSA for 1 h at room temperature. Membranes were probed with HRP-conjugated P4D1 anti-ubiquitin antibody and anti-GST antibody.

### Functional Analysis of the Interactome Dataset by DAVID Bioinformatic Resources

For the functional analysis of metabolic proteins enriched in the OTUB1 N22A interactome over OTUB1 WT interactome the online tool DAVID Bioinformatics Resources 6.7 (http://david.abcc.ncifcrf.gov/) was used [56,57]. Functional annotation clustering was performed using default settings.

### Establishment of Stable HEK293 OTUB1 Overexpression and Rescue Cell Lines

Control plasmids and plasmids encoding FLAG-OTUB1 WT or FLAG-OTUB1 N22A were linearized by digestion with PvuI (Thermo Scientific) and transiently transfected into HEK293 WT cells. Cells were selected with 1 mg/ml G418 for 4 wk.

An expression vector encoding a short hairpin RNA (shRNA) sequence targeting the 3′UTR of human OTUB1 (Sigma, TRCN0000273238) and a non-targeting shRNA (shControl) (Sigma, SHC016) were purchased from Sigma. Lentiviral particles for shOTUB1 and shControl were produced in HEK293T cells using the Vira-Power lentiviral expression vector system according to the manufacturer’s instructions (Invitrogen). HEK293 cells overexpressing FLAG-OTUB WT or N22A or control cells were infected with shOTUB1 or shControl lentiviral particles followed by selection with 2.5 μg/ml puromycin for 4 wk.

### Primer and Peptide Sequences

Primers designed for the site-directed mutagenesis of OTUB1 were as follows:

Forward primer 5′-AGGCCAGACAGGCAACACCTTCGGAGTCGCTGC-3′

Reverse primer 5′-GCAGCGACTCCGAAGGTGTTGCCTGTCTGGCCT-3′

Primers designed for the cloning of OTUB1 WT and N22A into pENTR4:

Forward primer 5′-ACGTCCATGGCGGCGGAGGAACCTCAGCA-3′

Reverse primer 5′-ACGTCTCGAGCTATTTGTAGAGGATATCGT-3′

Peptide sequences of peptides used in the in vitro hydroxylation assay:

Human HIF1 N803: DESGLPQLTSYDCEVNAPI

Murine Notch N2012: VEGMLEDLINSHADVNAVDD

Human OTUB1 N22: QQQKQEPLGSDSEGVNCLAYDEAIMAQQDRIQQE

Human OTUB1 N22A: QQQKQEPLGSDSEGVACLAYDEAIMAQQDRIQQE

### Statistical Analysis

For the analysis of statistical significance one-way ANOVA followed by Tukey test was applied for comparisons of more than two different datasets. For the comparison of two different datasets, unpaired Student’s *t* test was applied. *P*-values < 0.05 were considered statistically significant.

## Supporting Information

S1 Data Numerical data used in preparation of Figs 1B, 1D, 2A, 2C, 2D, 2F, 3C, 3D, 5B, 6B, 6D, 6F, S1B, S1D, S4A, and S6D. (XLSX)

S2 Data Dataset of the investigation of the OTUB1 WT and OTUB1 N22A interactome. Peptides assigned to several proteins are presented together with these assigned proteins in one row. Numerical data used in preparation of S1 Table.
(XLSX)

S1 Fig FIH-dependent regulation of cellular metabolism is dependent on its catalytic activity and the presence of OTUB1. **(A)** Western blot analysis of the impact of overexpression of the FIH catalytically dead mutant (H199A) on phosphorylation of AMPKα on T172 in HEK293 cells. Cells were transfected with either empty vector or FIH-V5 for 24 h prior to media change. Phospho-AMPKα, total AMPKα, β-actin and V5-FIH expression was assessed for up to 24 h following the addition of fresh medium by western blot. **(B)** Densitometric analysis of AMPKα phosphorylation at the time point 0 h. **(C)** Analysis of the impact of OTUB1 knockdown on FIH overexpression induced phosphorylation of AMPKα (T172) in HEK293 cells. Cells were transfected with control siRNA (siNT) or siOTUB1 for 24 h prior to the transfection of either an empty vector or pFLAG-OTUB1 WT for further 24 h. This was followed by media change and phospho-AMPKα, total AMPKα, β-actin and V5-FIH expression was assessed for up to 24 h by western blot. **(D)** Densitometric analysis of AMPKα phosphorylation at the time point 0 h. Data are presented as a representative blot or mean densitometric analysis derived from *n* = 4 independent experiments. The underlying data of panels B and D can be found in S1 Data.
(TIF)

S2 Fig Establishment of transient overexpression and knockdown of OTUB1 and FIH. **(A)** HEK293 cells in 12-well plates were transiently transfected with the indicated amounts of either empty vector (control), FLAG-HA-OTUB1, or FLAG-OTUB1 for 24 h prior to lysis. The relative OTUB1 overexpression levels were analyzed by western blot. **(B)** HEK293 cells in 12-well plates were transiently transfected with either control siRNA (siNT) or siRNA targeting the 3′-UTR of OTUB1 for 48 h prior to lysis. The transfection was performed at the indicated concentrations and with the indicated repeats. 300 ng of FLAG-HA-OTUB1 were transfected (low overexpression of OTUB1) 24 h prior to lysis. **(C)** HEK293 cells were transiently transfected in 12-well plates with either control siRNA (siNT) or siRNA targeting FIH for 48 h prior to lysis. The transfection was performed at the indicated concentrations and with the indicated repeats.
(TIF)

S3 Fig Analysis of N22 hydroxylation of OTUB1 in response to FIH overexpression or DMOG treatment. **(A), (B)** Extracted ion chromatograms showing peaks of OTUB1 peptides containing hydroxylated and non-hydroxylated asparagine 22. **(C)** Mass spectrometric analysis of non-hydroxylated and hydroxylated N22-containing OTUB1 peptides. **(D)** Tandem mass spectrometric analysis of N22 hydroxylation of OTUB1.
(TIF)

S4 Fig The impact of nutrient starvation and re-stimulation on OTUB1 N22 hydroxylation and the evolutionary conservation of the OTUB1 N22 hydroxylation site. **(A)** HEK293 cells were transiently transfected with FLAG-HA-OTUB1 and FIH-V5 for 24 h prior to nutrient starvation for 8 h or nutrient starvation (8 h) followed by re-stimulation with nutrient rich media for one additional hour. Control cells were incubated with nutrient rich media throughout the experiment. Following FLAG-specific immunoprecipitation the hydroxylation levels of OTUB1 N22 were analyzed using mass spectrometry. The detected hydroxylation peptide intensity of each sample was normalized to the overall amount of total OTUB1 intensity detected. The experiment was performed with three biological replicates and two technical replicates per sample. Data are represented as mean + SD. * *p* < 0.05, *** *p* < 0.001 by one-way ANOVA followed by Tukey post test. **(B)** Protein sequences of the OTUB1 protein of different species were downloaded from the Uniprot database (www.uniprot.org) and aligned with the multiple sequence alignment tool Clustal Omega (http://www.ebi.ac.uk/Tools/msa/clustalo/) [58,59]. Amino acid residues that are part of the FIH consensus sequence in human OTUB1 are highlighted in gray. The FIH-targeted N22 residue of human OTUB1 is highlighted with “*”. The underlying data of panel A can be found in S1 Data.
(TIF)

S5 Fig The impact of OTUB1 N22 hydroxylation on known OTUB1 interactors and the verification of its impact on the interaction with FIH. HEK293 cells were transfected in biological triplicates with pFIH-V5 and either empty vector (control), pFLAG-OTUB1 WT or pFLAG-OTUB1 N22A for 24 h prior to FLAG-specific immunoprecipitation. The precipitants were analyzed for associated proteins by mass spectrometry and the values were normalized to relative amounts of precipitated OTUB1. Interaction of FLAG-OTUB1 WT and FLAG-OTUB1 N22A with **(A)** Ubiquitin^#^, **(B)** UBE2N (UBC13), **(C)** UBE2D1 (UBCH5A)^§^, **(D)** UBE2D2 (UBCH5B), **(E)** UBE2D3 (UBCH5C), and **(F)** MSH2. # The peptides identified as ubiquitin were not unambiguously identified. It was not possible to distinguish between the following proteins: RPS27A, UBC, UBB, UBA52. § The peptide sequences identified could also belong to UBE2D4 according to the Uniprot (www.uniprot.org) entry with the identifier Q9UQL0. However, this entry has not been reviewed yet and shows a low annotation score. **(G)** HEK293 cells were transfected with FIH-V5 and either empty vector (control), FLAG-OTUB1 WT, or N22A for 24 h prior to lysis and FLAG-specific immunoprecipitation. The levels of protein overexpression and the efficacy of the (co-)immunoprecipitation were determined by western blot. Representative blot of *n* = 3 independent experiments.
The experiment shown in (A)–(F) was performed with three biological replicates and two technical replicates per sample. Data presented as mean + SD. * *p* < 0.05, ** *p* < 0.01, *** *p* < 0.001 by Student’s *t* test.
(TIF)

S6 Fig The OTUB1 N22A point mutation does not change OTUB1 enzymatic activity. **(A)** FLAG tagged WT and N22A OTUB1 were immunoprecipitated from stably transfected HEK293 cells and washed resins incubated with 600 nM K48-tetraubiquitin (K48-Ub_4_) at 37°C for the indicated time points. HEK293 cells stably transfected with an empty vector (Control) were used as control. DUB activity was measured by western blot for ubiquitin. We used 20 nM mammalian 26S proteasomes (26S) as a control for DUB activity. **(B)** The purity of recombinantly expressed GST-OTUB1 and GST-OTUB1 N22A following dialysis was measured by Coomassie staining. **(C)** Purified GST-OTUB1 WT and GST-OTUB1 N22A were incubated with 600 nM K48-Ub_4_ at 37°C for the times indicated and the DUB activity was measured by Western Blot for ubiquitin. **(D)** Densitometric analysis of the levels of the K48-linked chains. Data are presented as representative blot or as mean ± SEM of *n* = 3 independent experiments. The underlying data of panel D can be found in S1 Data.
(TIF)

S7 Fig Point mutation of asparagine 22 to alanine regulates the association of OTUB1 with proteins of different pathways. **(A)** Gene Ontology analysis for biological processes of proteins significantly enriched and increased by at least 2-fold in FLAG-OTUB1 WT over control. **(B)** Gene Ontology analysis for biological processes of the proteins significantly enriched and increased by at least 2-fold in FLAG-OTUB1 N22A over control. The analyses were performed using Panther database (www.pantherdb.org).
(TIF)

S8 Fig Establishment of stably transfected cells lines. HEK293 cells were stably transfected with either empty vector (control), FLAG-OTUB1 WT or FLAG-OTUB1 N22A alone or with the combination of control and a non-targeting shRNA (shControl), control and a shRNA targeting the 3′UTR of OTUB1 (shOTUB1), FLAG-OTUB1 WT and shOTUB1, or FLAG-OTUB1 N22A and shOTUB1. OTUB1 overexpression and knockdown were determined by western blot.
(TIF)

S1 Table DAVID Functional annotation of metabolic proteins significantly enriched in the OTUB1 N22A over WT interactome. Proteins significantly enriched in OTUB1 N22A co-precipitants over OTUB1 WT co-precipitants were analyzed with DAVID Bioinformatics Resources (http://david.abcc.ncifcrf.gov/) for functional annotation clustering. Listed are the obtained clusters and proteins for metabolic pathways.
(DOCX)

## Abbreviations

ARD: ankyrin-repeat domain
CAD: C-terminal transactivation domain
CHX: cycloheximide
DUB: deubiquitinase
FIH: factor inhibiting HIF
HIF: hypoxia-inducible factor
MS/MS: tandem mass spectrometry
N22A: point mutation of asparagine 22 to alanine
O_2_: molecular oxygen
OTUB1: ovarian tumor domain containing ubiquitin aldehyde binding protein 1
PHD: prolyl-4-hydroxylase
shRNA: short hairpin RNA
siFIH: siRNA targeting FIH
siNT: non-targeting siRNA
siRNA: short interfering RNA
VHL: von Hippel-Lindau protein
WT: wild type
