# Supplementary figures and images for: FIH Regulates Cellular Metabolism through Hydroxylation of the Deubiquitinase OTUB1
Source: PLoS Biol. 2016 Jan 11;14(1):e1002347. doi: 10.1371/journal.pbio.1002347 (PMC4709136; doi:10.1371/journal.pbio.1002347)

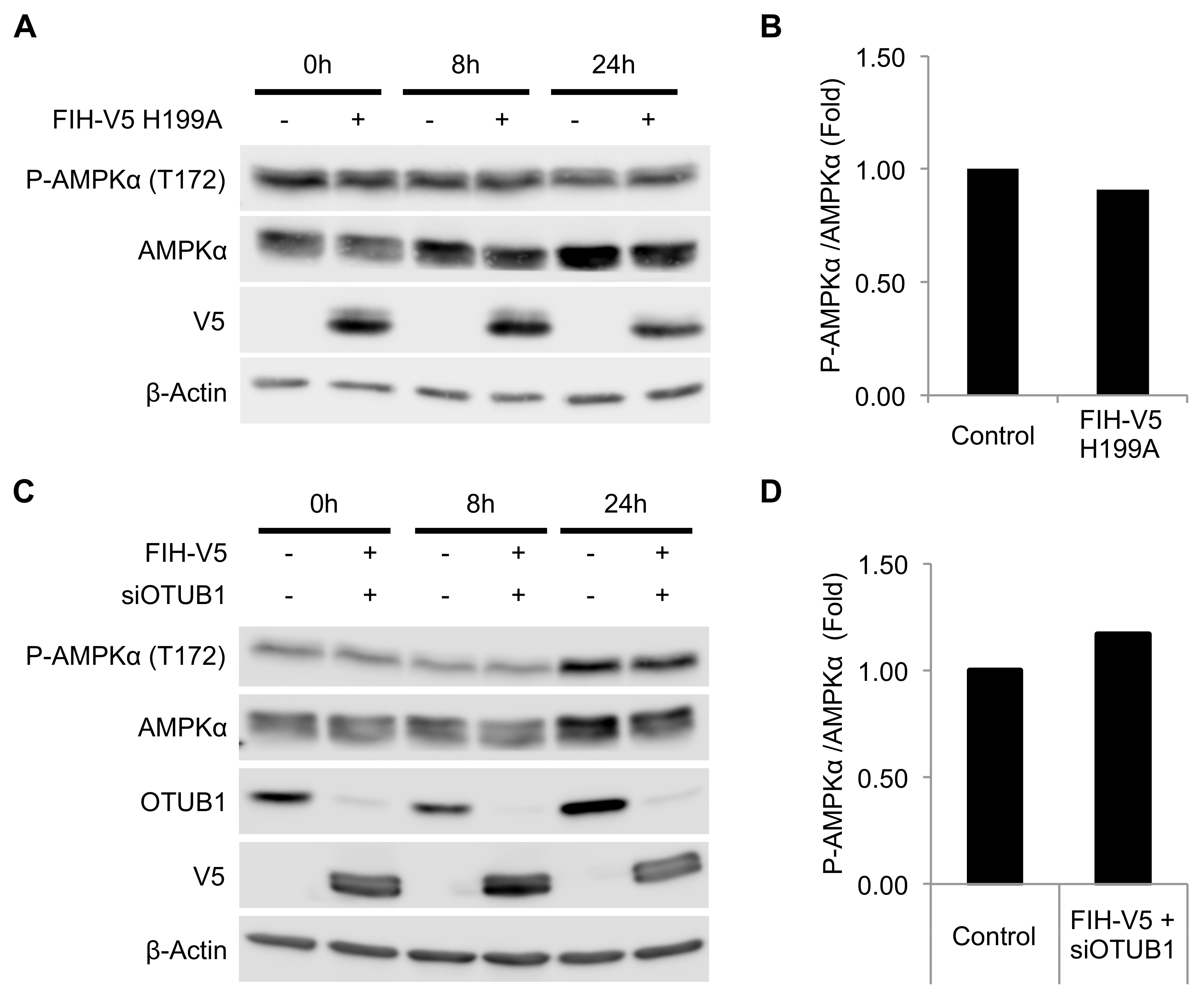

Supplement: S1 Fig — (A) Western blot analysis of the impact of overexpression of the FIH catalytically dead mutant (H199A) on phosphorylation of AMPKα on T172 in HEK293 cells. Cells were transfected with either empty vector or FIH-V5 for 24 h prior to media change. Phospho-AMPKα, total AMPKα, β-actin and V5-FIH expression was assessed for up to 24 h following the addition of fresh medium by western blot. (B) Densitometric analysis of AMPKα phosphorylation at the time point 0 h. (C) Analysis of the impact of OTUB1 knockdown on FIH overexpression induced phosphorylation of AMPKα (T172) in HEK293 cells. Cells were transfected with control siRNA (siNT) or siOTUB1 for 24 h prior to the transfection of either an empty vector or pFLAG-OTUB1 WT for further 24 h. This was followed by media change and phospho-AMPKα, total AMPKα, β-actin and V5-FIH expression was assessed for up to 24 h by western blot. (D) Densitometric analysis of AMPKα phosphorylation at the time point 0 h. Data are presented as a representative blot or mean densitometric analysis derived from n = 4 independent experiments. The underlying data of panels B and D can be found in S1 Data. (TIF) [file pbio.1002347.s003.tif]

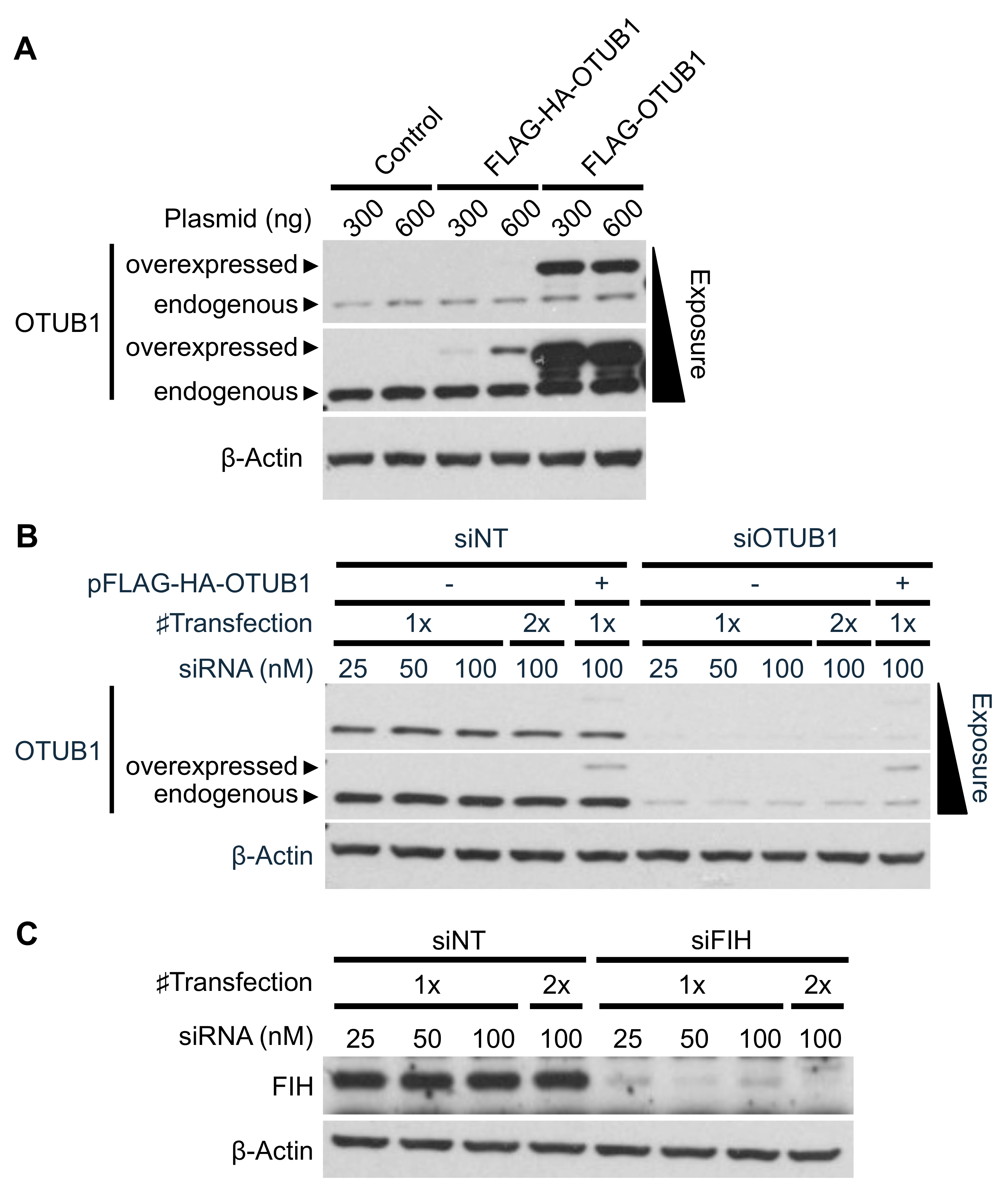

Supplement: S2 Fig — (A) HEK293 cells in 12-well plates were transiently transfected with the indicated amounts of either empty vector (control), FLAG-HA-OTUB1, or FLAG-OTUB1 for 24 h prior to lysis. The relative OTUB1 overexpression levels were analyzed by western blot. (B) HEK293 cells in 12-well plates were transiently transfected with either control siRNA (siNT) or siRNA targeting the 3′-UTR of OTUB1 for 48 h prior to lysis. The transfection was performed at the indicated concentrations and with the indicated repeats. 300 ng of FLAG-HA-OTUB1 were transfected (low overexpression of OTUB1) 24 h prior to lysis. (C) HEK293 cells were transiently transfected in 12-well plates with either control siRNA (siNT) or siRNA targeting FIH for 48 h prior to lysis. The transfection was performed at the indicated concentrations and with the indicated repeats. (TIF) [file pbio.1002347.s004.tif]

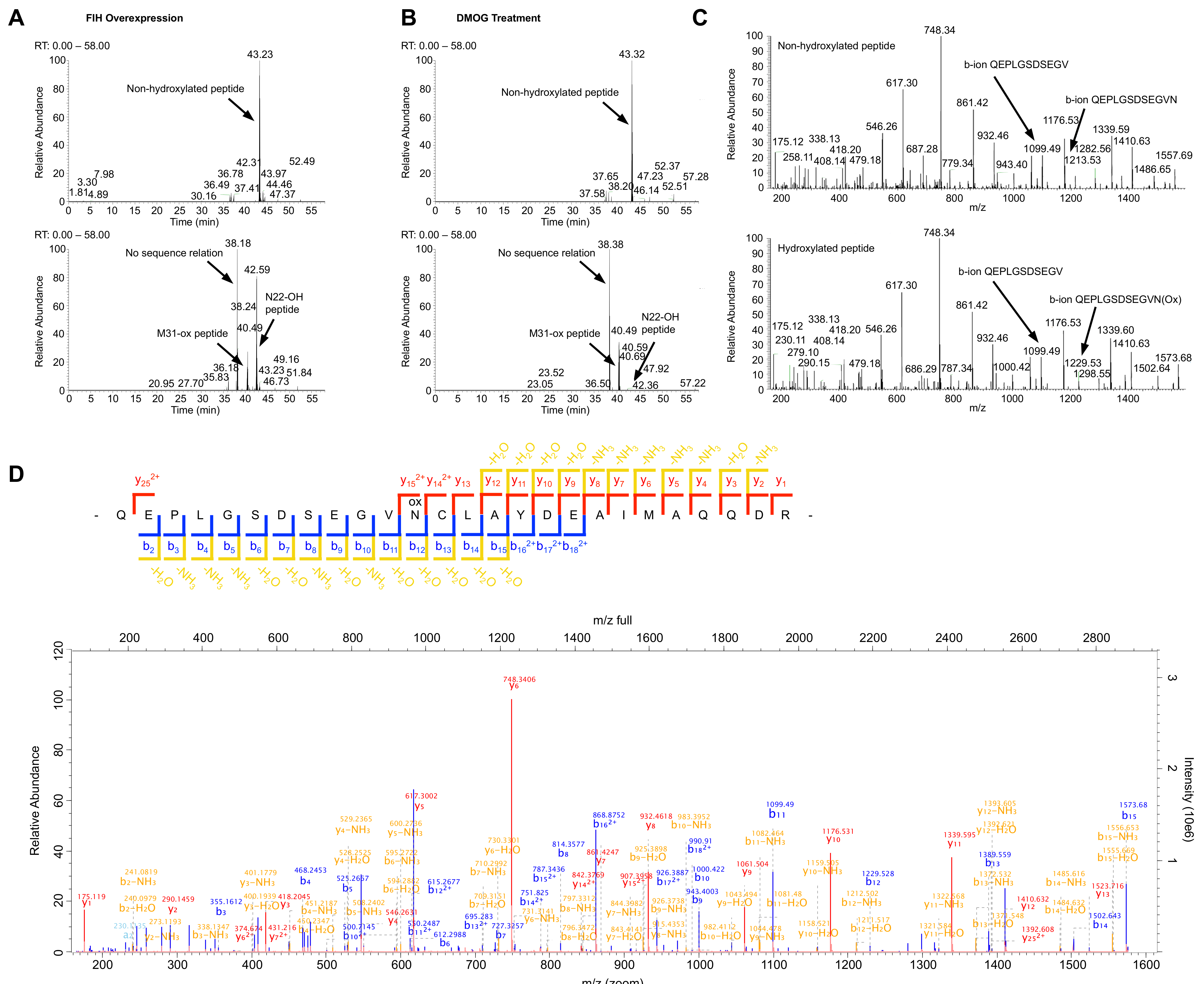

Supplement: S3 Fig — (A), (B) Extracted ion chromatograms showing peaks of OTUB1 peptides containing hydroxylated and non-hydroxylated asparagine 22. (C) Mass spectrometric analysis of non-hydroxylated and hydroxylated N22-containing OTUB1 peptides. (D) Tandem mass spectrometric analysis of N22 hydroxylation of OTUB1. (TIF) [file pbio.1002347.s005.tif]

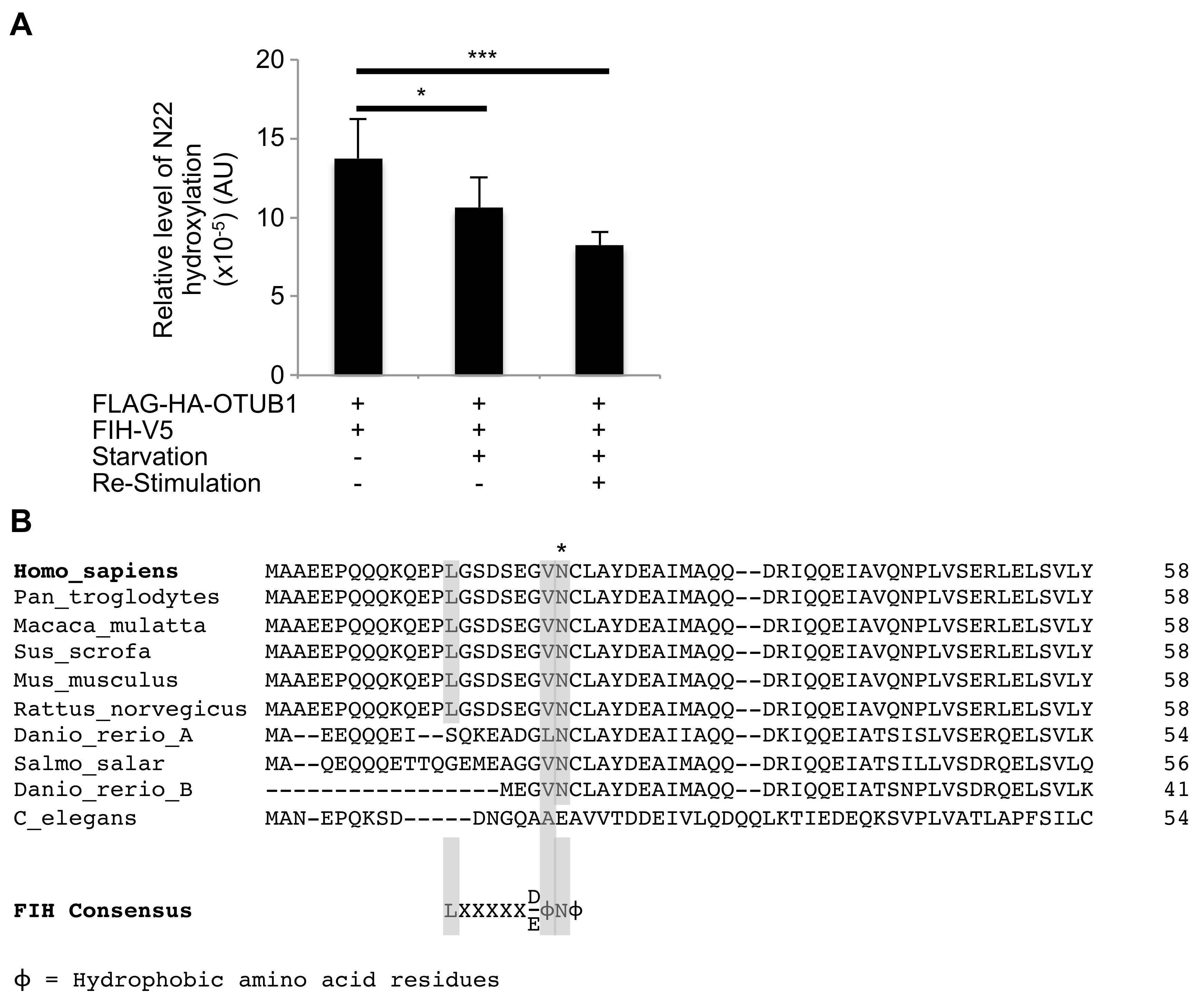

Supplement: S4 Fig — (A) HEK293 cells were transiently transfected with FLAG-HA-OTUB1 and FIH-V5 for 24 h prior to nutrient starvation for 8 h or nutrient starvation (8 h) followed by re-stimulation with nutrient rich media for one additional hour. Control cells were incubated with nutrient rich media throughout the experiment. Following FLAG-specific immunoprecipitation the hydroxylation levels of OTUB1 N22 were analyzed using mass spectrometry. The detected hydroxylation peptide intensity of each sample was normalized to the overall amount of total OTUB1 intensity detected. The experiment was performed with three biological replicates and two technical replicates per sample. Data are represented as mean + SD. * p < 0.05, *** p < 0.001 by one-way ANOVA followed by Tukey post test. (B) Protein sequences of the OTUB1 protein of different species were downloaded from the Uniprot database (www.uniprot.org) and aligned with the multiple sequence alignment tool Clustal Omega (http://www.ebi.ac.uk/Tools/msa/clustalo/) [58,59]. Amino acid residues that are part of the FIH consensus sequence in human OTUB1 are highlighted in gray. The FIH-targeted N22 residue of human OTUB1 is highlighted with “*”. The underlying data of panel A can be found in S1 Data. (TIF) [file pbio.1002347.s006.tif]

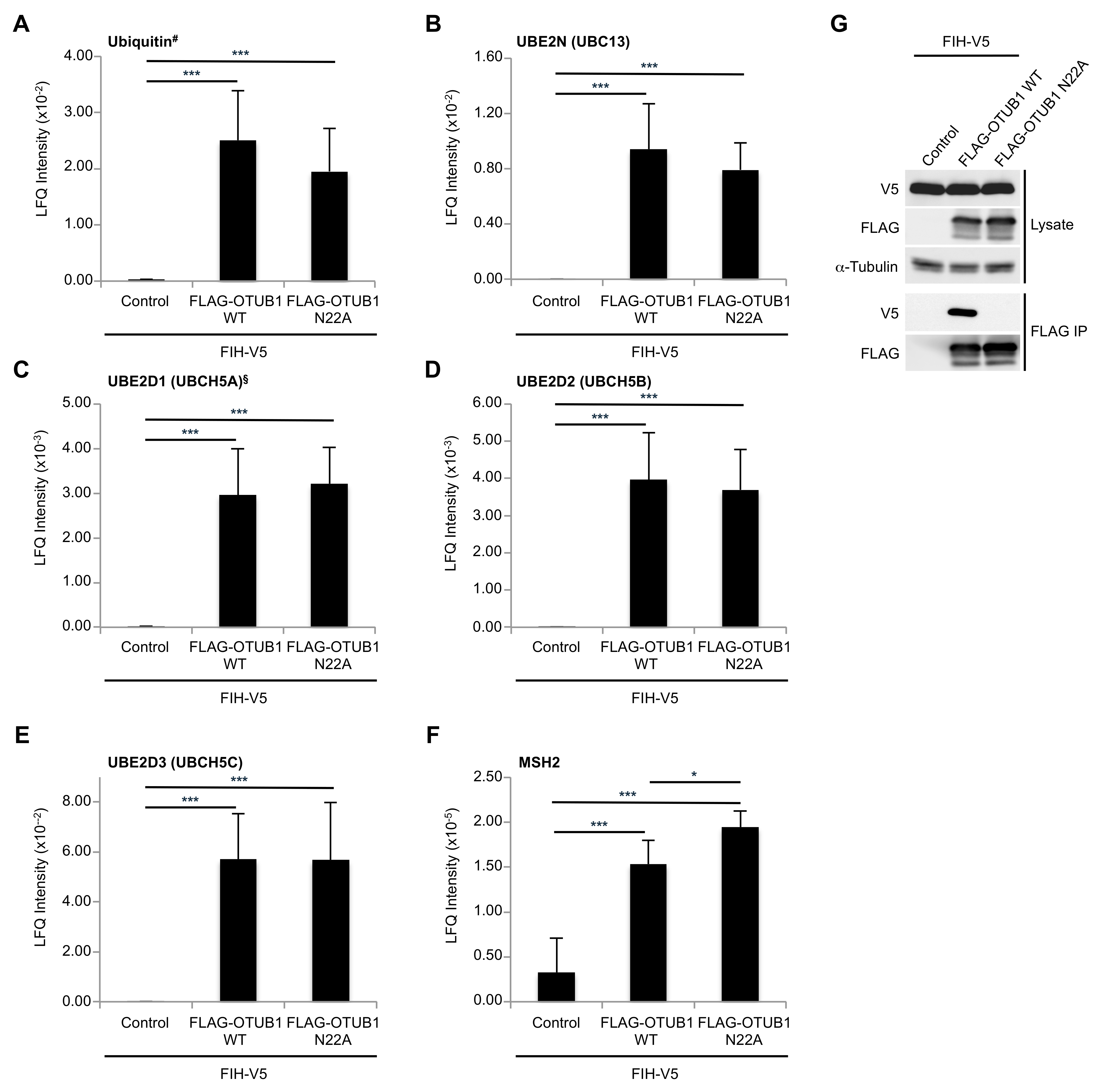

Supplement: S5 Fig — HEK293 cells were transfected in biological triplicates with pFIH-V5 and either empty vector (control), pFLAG-OTUB1 WT or pFLAG-OTUB1 N22A for 24 h prior to FLAG-specific immunoprecipitation. The precipitants were analyzed for associated proteins by mass spectrometry and the values were normalized to relative amounts of precipitated OTUB1. Interaction of FLAG-OTUB1 WT and FLAG-OTUB1 N22A with (A) Ubiquitin#, (B) UBE2N (UBC13), (C) UBE2D1 (UBCH5A)§, (D) UBE2D2 (UBCH5B), (E) UBE2D3 (UBCH5C), and (F) MSH2. # The peptides identified as ubiquitin were not unambiguously identified. It was not possible to distinguish between the following proteins: RPS27A, UBC, UBB, UBA52. § The peptide sequences identified could also belong to UBE2D4 according to the Uniprot (www.uniprot.org) entry with the identifier Q9UQL0. However, this entry has not been reviewed yet and shows a low annotation score. (G) HEK293 cells were transfected with FIH-V5 and either empty vector (control), FLAG-OTUB1 WT, or N22A for 24 h prior to lysis and FLAG-specific immunoprecipitation. The levels of protein overexpression and the efficacy of the (co-)immunoprecipitation were determined by western blot. Representative blot of n = 3 independent experiments. The experiment shown in (A)–(F) was performed with three biological replicates and two technical replicates per sample. Data presented as mean + SD. * p < 0.05, ** p < 0.01, *** p < 0.001 by Student’s t test. (TIF) [file pbio.1002347.s007.tif]

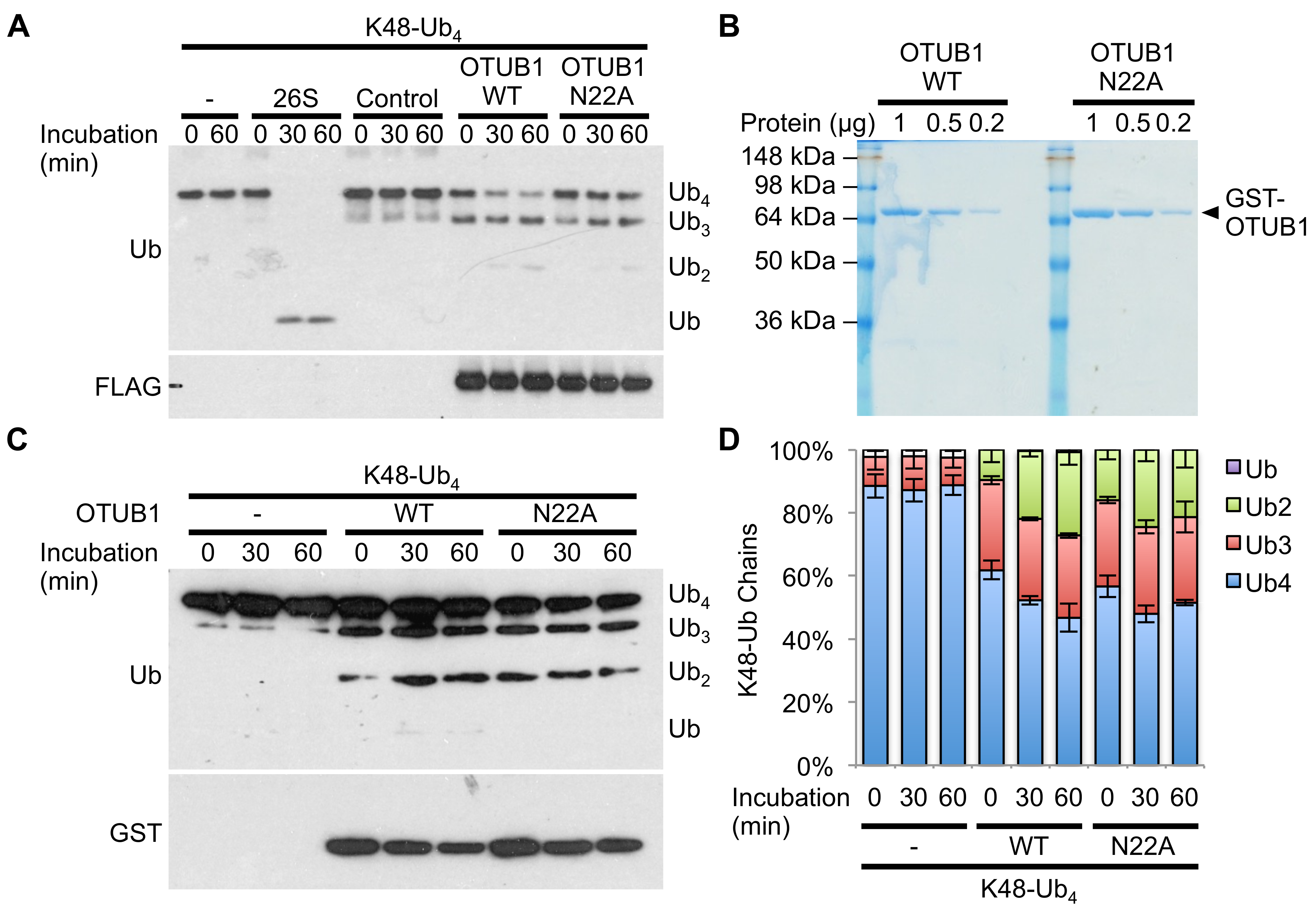

Supplement: S6 Fig — (A) FLAG tagged WT and N22A OTUB1 were immunoprecipitated from stably transfected HEK293 cells and washed resins incubated with 600 nM K48-tetraubiquitin (K48-Ub4) at 37°C for the indicated time points. HEK293 cells stably transfected with an empty vector (Control) were used as control. DUB activity was measured by western blot for ubiquitin. We used 20 nM mammalian 26S proteasomes (26S) as a control for DUB activity. (B) The purity of recombinantly expressed GST-OTUB1 and GST-OTUB1 N22A following dialysis was measured by Coomassie staining. (C) Purified GST-OTUB1 WT and GST-OTUB1 N22A were incubated with 600 nM K48-Ub4 at 37°C for the times indicated and the DUB activity was measured by Western Blot for ubiquitin. (D) Densitometric analysis of the levels of the K48-linked chains. Data are presented as representative blot or as mean ± SEM of n = 3 independent experiments. The underlying data of panel D can be found in S1 Data. (TIF) [file pbio.1002347.s008.tif]

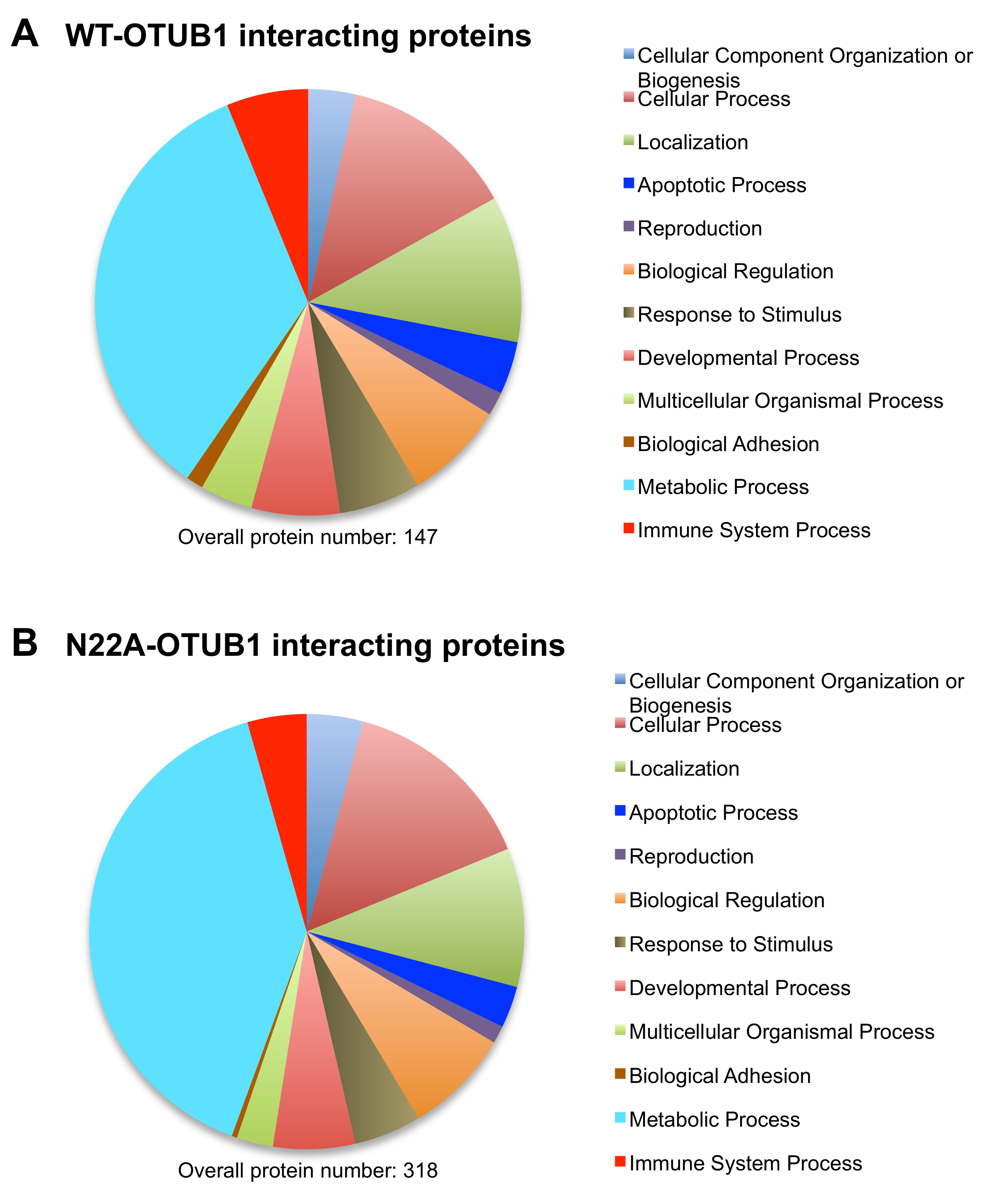

Supplement: S7 Fig — (A) Gene Ontology analysis for biological processes of proteins significantly enriched and increased by at least 2-fold in FLAG-OTUB1 WT over control. (B) Gene Ontology analysis for biological processes of the proteins significantly enriched and increased by at least 2-fold in FLAG-OTUB1 N22A over control. The analyses were performed using Panther database (www.pantherdb.org). (TIF) [file pbio.1002347.s009.tif]

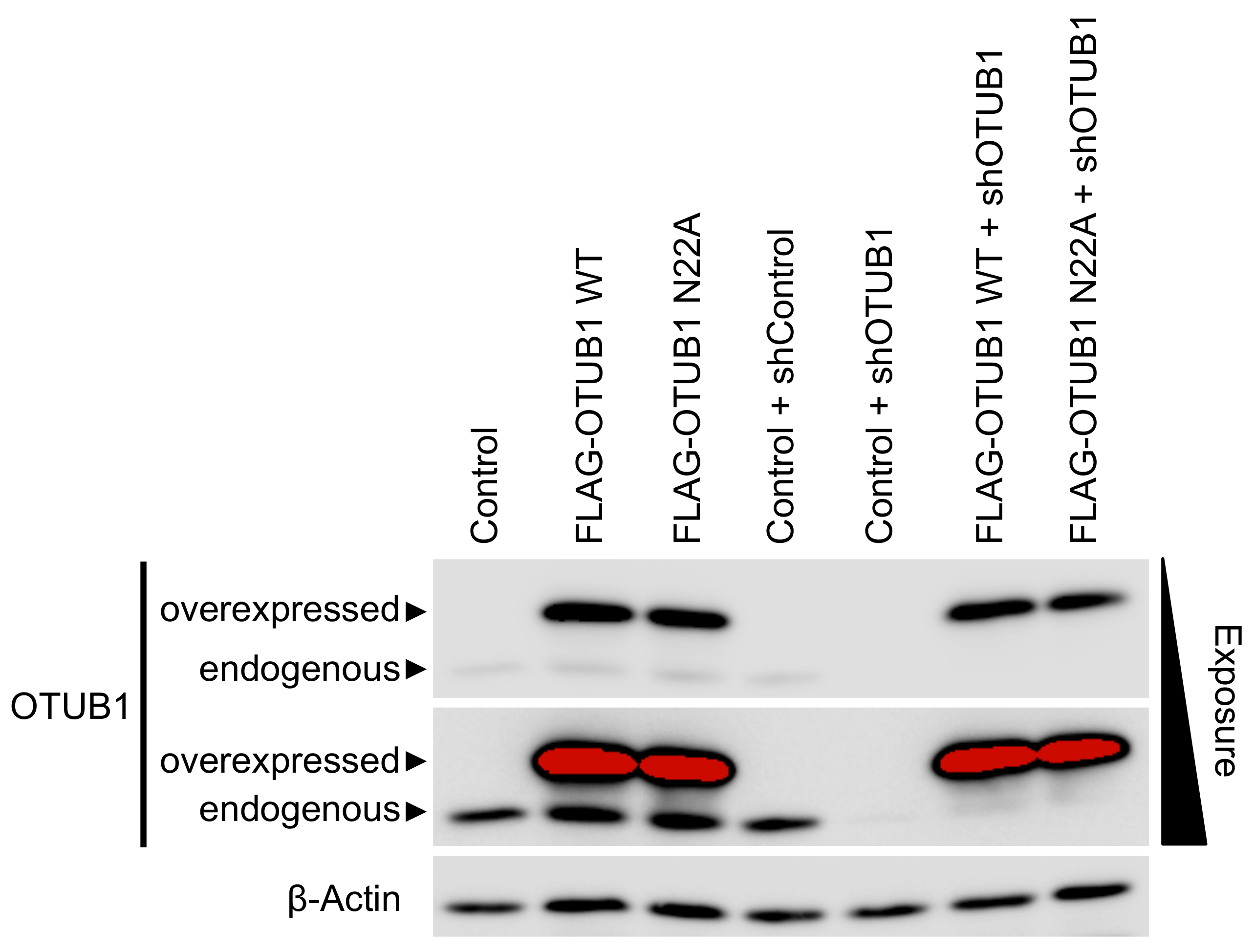

Supplement: S8 Fig — HEK293 cells were stably transfected with either empty vector (control), FLAG-OTUB1 WT or FLAG-OTUB1 N22A alone or with the combination of control and a non-targeting shRNA (shControl), control and a shRNA targeting the 3′UTR of OTUB1 (shOTUB1), FLAG-OTUB1 WT and shOTUB1, or FLAG-OTUB1 N22A and shOTUB1. OTUB1 overexpression and knockdown were determined by western blot. (TIF) [file pbio.1002347.s010.tif]
