# Supplementary material for: FIH Regulates Cellular Metabolism through Hydroxylation of the Deubiquitinase OTUB1
Source: PLoS Biol. 2016 Jan 11;14(1):e1002347. doi: 10.1371/journal.pbio.1002347 (PMC4709136; doi:10.1371/journal.pbio.1002347)
Supplement: S1 Table — Proteins significantly enriched in OTUB1 N22A co-precipitants over OTUB1 WT co-precipitants were analyzed with DAVID Bioinformatics Resources (http://david.abcc.ncifcrf.gov/) for functional annotation clustering. Listed are the obtained clusters and proteins for metabolic pathways. (DOCX) [file pbio.1002347.s011.docx]

**S1 Table**

| Pathway | ID | | Gene Name | Ratio (over control) |
| --- | --- | --- | --- | --- |
| Glycolysis | ALDOA | | Aldolase A, fructose-bisphosphate | 3.65 |
|  | DLD | | Dihydrolipoamide dehydrogenase | 3.03 |
|  | ENO1 | | Enolase 1, (alpha) | 3.08 |
|  | GAPDH | | Glyceraldehyde-3-phosphate Dehydrogenase-like 6; hypothetical protein LOC100133042; glyceraldehyde-3-phosphate dehydrogenase | 2.81 |
|  | LDHA | | Lactate dehydrogenase A | 3.54 |
|  | LDHB | | Lactate dehydrogenase B | 2.82 |
|  | PGK1 | | Phosphoglycerate kinase 1 | 2.89 |
|  | PGAM1 | | Phosphoglycerate mutase 1 (brain) | 3.82 |
|  | PGAM2 | | Phosphoglycerate mutase 2 (muscle) | 3.82 |
|  | PKM2 | | Similar to Pyruvate kinase, isozymes M1/M2 (Pyruvate kinase muscle isozyme) (Cytosolic thyroid hormone-binding protein) (CTHBP) (THBP1); pyruvate kinase, muscle | 2.66 |
| TCA Cycle | ACLY | ATP citrate lyase | | 4.08 |
|  | CS | Citrate synthase | | 3.80 |
|  | DLD | Dihydrolipoamide dehydrogenase | | 3.03 |
| Oxidative Phosphorylation | ATP5F1 | ATP synthase, H+ transporting, mitochondrial F0 complex, subunit B1 | | 4.43 |
|  | ATP5A1 | ATP synthase, H+ transporting, mitochondrial F1 complex, alpha subunit 1, cardiac muscle | | 2.76 |
|  | ATP5B | ATP synthase, H+ transporting, mitochondrial F1 complex, beta polypeptide | | 2.57 |
|  | ATP6V1A | ATPase, H+ transporting, lysosomal 70kDa, V1 subunit A | | 4.02 |
|  | COX5A | Cytochrome c oxidase subunit Va | | 2.92 |
|  | UQCRC1 | Ubiquinol-cytochrome c reductase core protein I | | 2.95 |
|  | UQCRC2 | Ubiquinol-cytochrome c reductase core protein II | | 3.10 |
|  | UQCRFS1 | Ubiquinol-cytochrome c reductase, Rieske iron-sulfur polypeptide-like 1; ubiquinol-cytochrome c reductase, Rieske iron-sulfur polypeptide 1 | | 6.61 |
|  | UQCRFS1P1 | Ubiquinol-cytochrome c reductase Rieske iron-sulfur subunit pseudogene 1 | | 6.61 |
| Purine Metabolism | ATIC | 5-aminoimidazole-4-carboxamide ribonucleotide formyltransferase/IMP cyclohydrolase | | 3.65 |
|  | AK2 | Adenylate kinase 2 | | 3.85 |
|  | ADSS | Adenylosuccinate synthase | | 10.52 |
|  | NME2, NME1-NME2, NME1 | Non-metastatic cells 1, protein (NM23A) expressed in; NME1-NME2 readthrough transcript; non-metastatic cells 2, protein (NM23B) expressed in | | 2.24 |
|  | PPAT | Phosphoribosyl pyrophosphate amidotransferase | | 4.20 |
|  | PKM2 | Similar to Pyruvate kinase, isozymes M1/M2 (Pyruvate kinase muscle isozyme) (Cytosolic thyroid hormone-binding protein) (CTHBP) (THBP1); pyruvate kinase, muscle | | 2.66 |
| Pyruvate Metabolism | DLD | Dihydrolipoamide dehydrogenase | | 3.03 |
|  | LDHA | Lactate dehydrogenase A | | 3.54 |
|  | LDHB | Lactate dehydrogenase B | | 2.82 |
|  | PKM2 | Similar to Pyruvate kinase, isozymes M1/M2 (Pyruvate kinase muscle isozyme) (Cytosolic thyroid hormone-binding protein) (CTHBP) (THBP1); pyruvate kinase, muscle | | 2.66 |
|  | ACAT1 | Acetyl-Coenzyme A acetyltransferase 1 | | 5.05 |
|  | ACAT2 | Acetyl-Coenzyme A acetyltransferase 2 | | 4.06 |
| Propanoate Metabolism | LDHA | Lactate dehydrogenase A | | 3.54 |
|  | LDHB | Lactate dehydrogenase B | | 2.82 |
|  | ACAT1 | Acetyl-Coenzyme A acetyltransferase 1 | | 5.05 |
|  | ACAT2 | Acetyl-Coenzyme A acetyltransferase 2 | | 4.06 |
| Glutathione Metabolism | GSTM2 | Glutathione S-transferase mu 2 (muscle) | | 3.75 |
|  | GSTM3 | Glutathione S-transferase mu 3 (brain) | | 3.75 |
|  | GSTM5 | Glutathione S-transferase mu 5 | | 3.75 |
|  | GSTP1 | Glutathione S-transferase pi 1 | | 3.33 |
|  | SRM | Spermidine synthase | | 4.98 |
